# Supplementary figures and images for: Accurate genetic and environmental covariance estimation with composite likelihood in genome-wide association studies
Source: PLoS Genet. 2021 Jan 4;17(1):e1009293. doi: 10.1371/journal.pgen.1009293 (PMC7808654; doi:10.1371/journal.pgen.1009293)

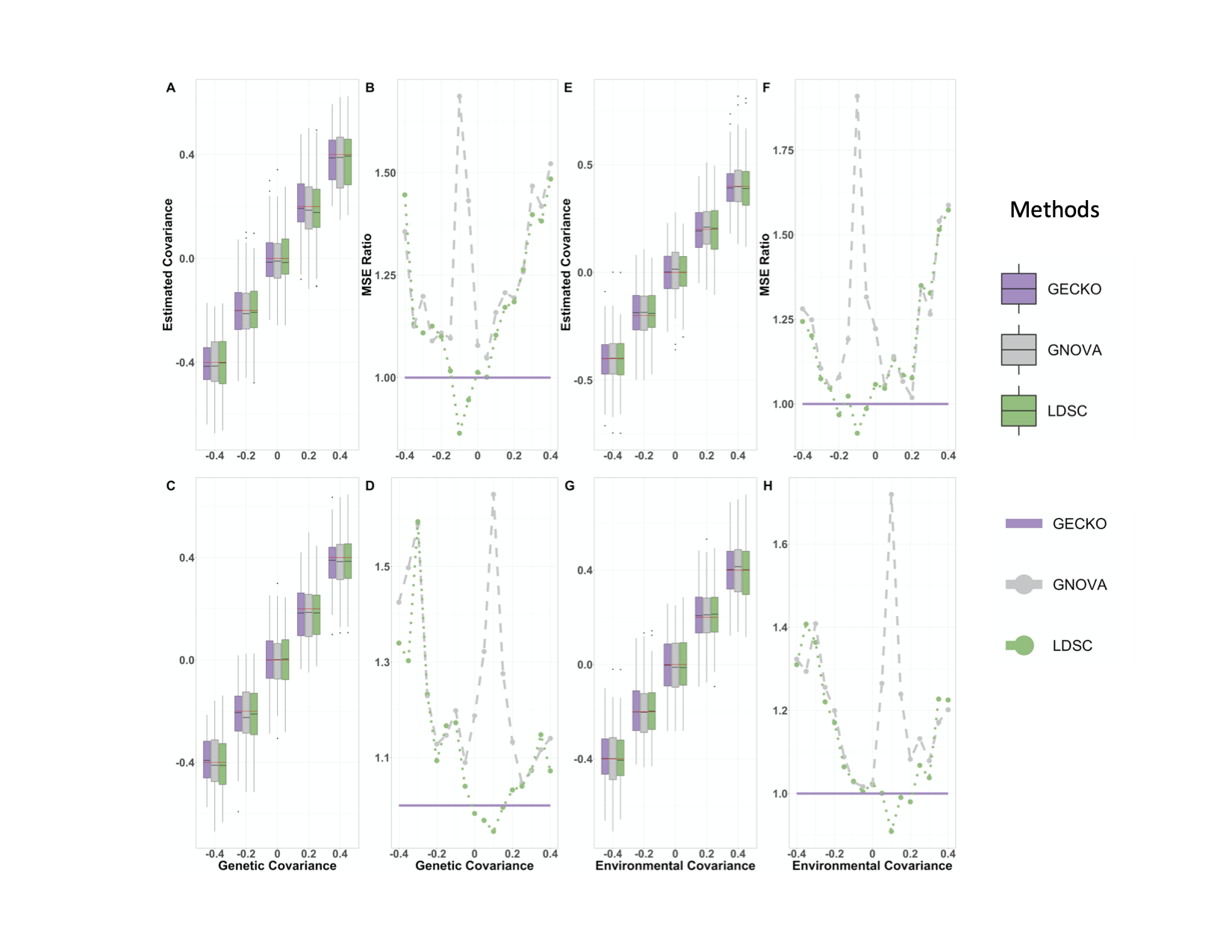

Supplement: S1 Fig — Compared methods include GECKO (purple), GNOVA (grey), and LDSC (green). Results are shown for the one study design with positive genetic or environmental covariance (first row: A, B, E, F) and the one study design with negative genetic or environmental covariance (second row: C, D, G, H). Boxplots display estimated genetic covariances (A, C) and environmental covariances (E, G) on y-axis versus the true covariances on x-axis across simulation replicates. Estimation accuracy is measured by the ratio of mean square errors (MSE), which contrast the MSE from GNOVA or LDSC with respect to GECKO, across various true covariances on x-axis, for genetic (B, D) and environmental covariances (F, H). An MSE ratio below one suggests that GECKO performs worse than the other method; above one otherwise. (TIF) [file pgen.1009293.s001.tif]

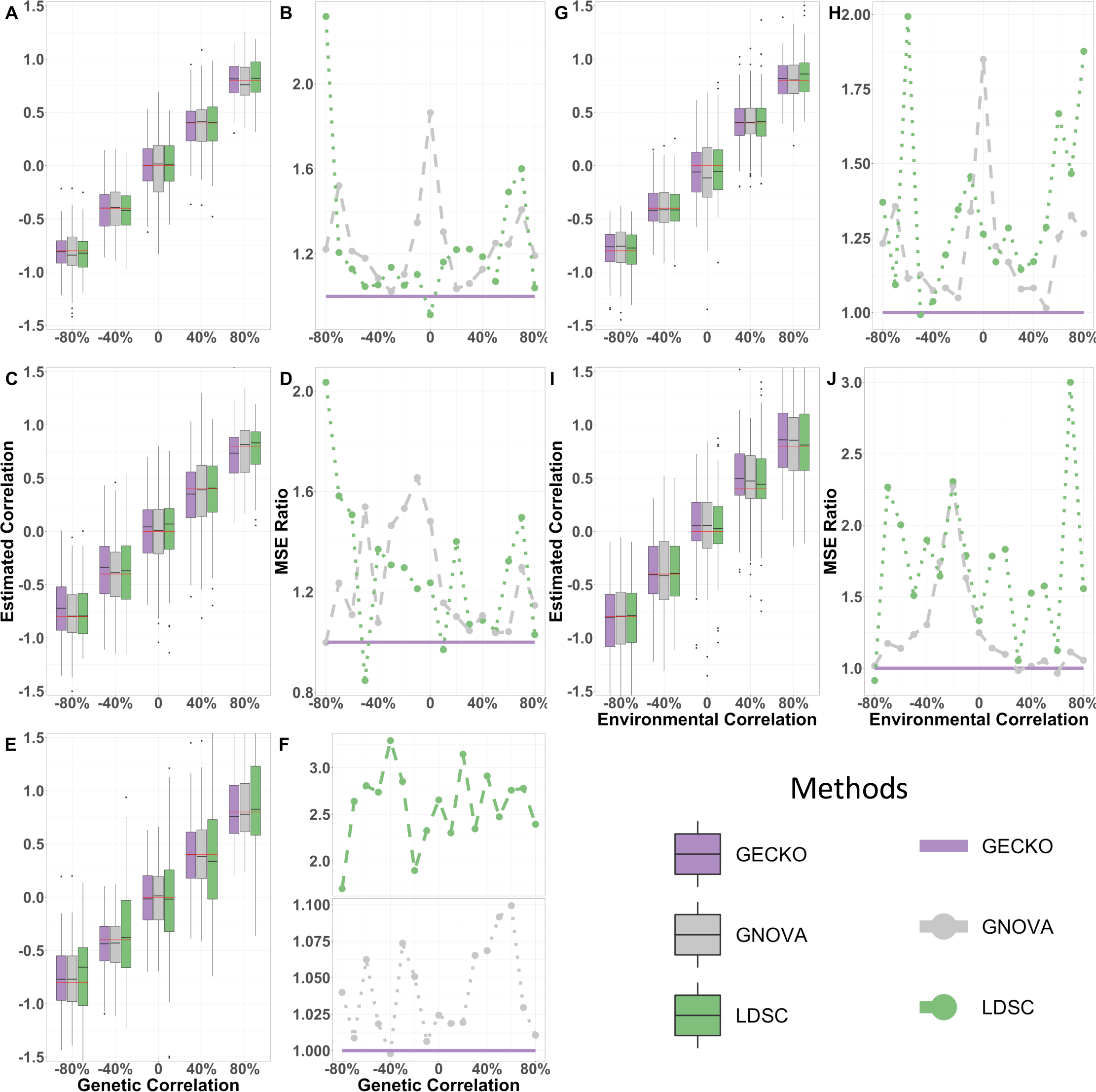

Supplement: S2 Fig — Compared methods include GECKO (purple), GNOVA (grey), and LDSC (green). Results are shown for the one study design (first row: A, B, G, H), two partially overlapped study design (second row: C, D, I, J), and two separate study design (third row: E, F). Boxplots display estimated genetic correlation (A, C, E) and environmental correlation (G, I) on y-axis versus the true covariances on x-axis across simulation replicates. Estimation accuracy is measured by the ratio of mean square errors (MSE), which contrast the MSE from GNOVA or LDSC with respect to GECKO, across various true correlation on x-axis, for genetic (B, D, F) and environmental (H, J) correlation. An MSE ratio below one suggests that GECKO performs worse than the other method; above one otherwise. (TIF) [file pgen.1009293.s002.tif]

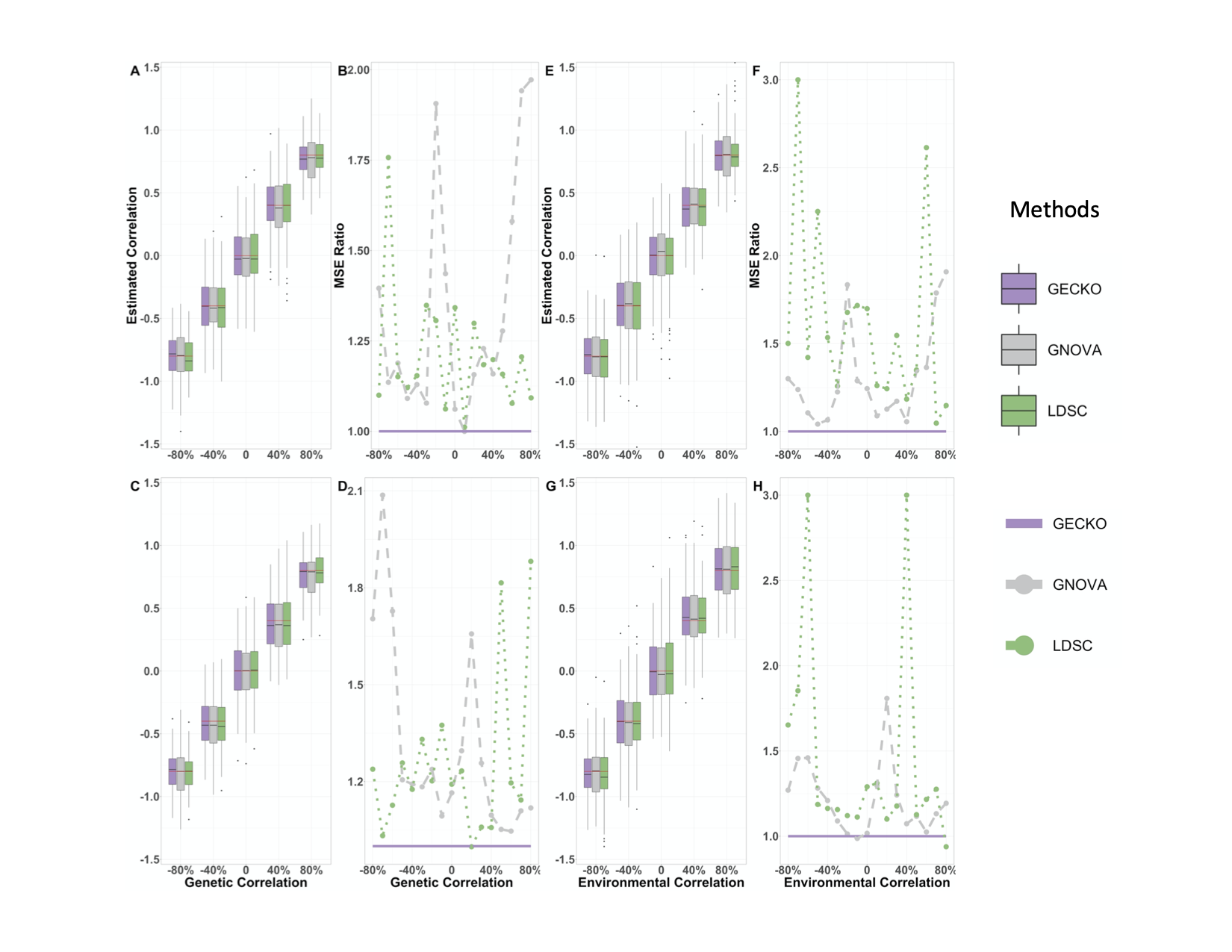

Supplement: S3 Fig — Compared methods include GECKO (purple), GNOVA (grey), and LDSC (green). Results are shown for the one study design with positive genetic or environmental correlation (first row: A, B, E, F) and the one study design with negative genetic or environmental correlation (second row: C, D, G, H). Boxplots display estimated genetic correlation (A, C) and environmental correlation (E, G) on y-axis versus the true correlation on x-axis across simulation replicates. Estimation accuracy is measured by the ratio of mean square errors (MSE), which contrast the MSE from GNOVA or LDSC with respect to GECKO, across various true correlation on x-axis, for genetic (B, D) and environmental correlation (F, H). An MSE ratio below one suggests that GECKO performs worse than the other method; above one otherwise. (TIF) [file pgen.1009293.s003.tif]

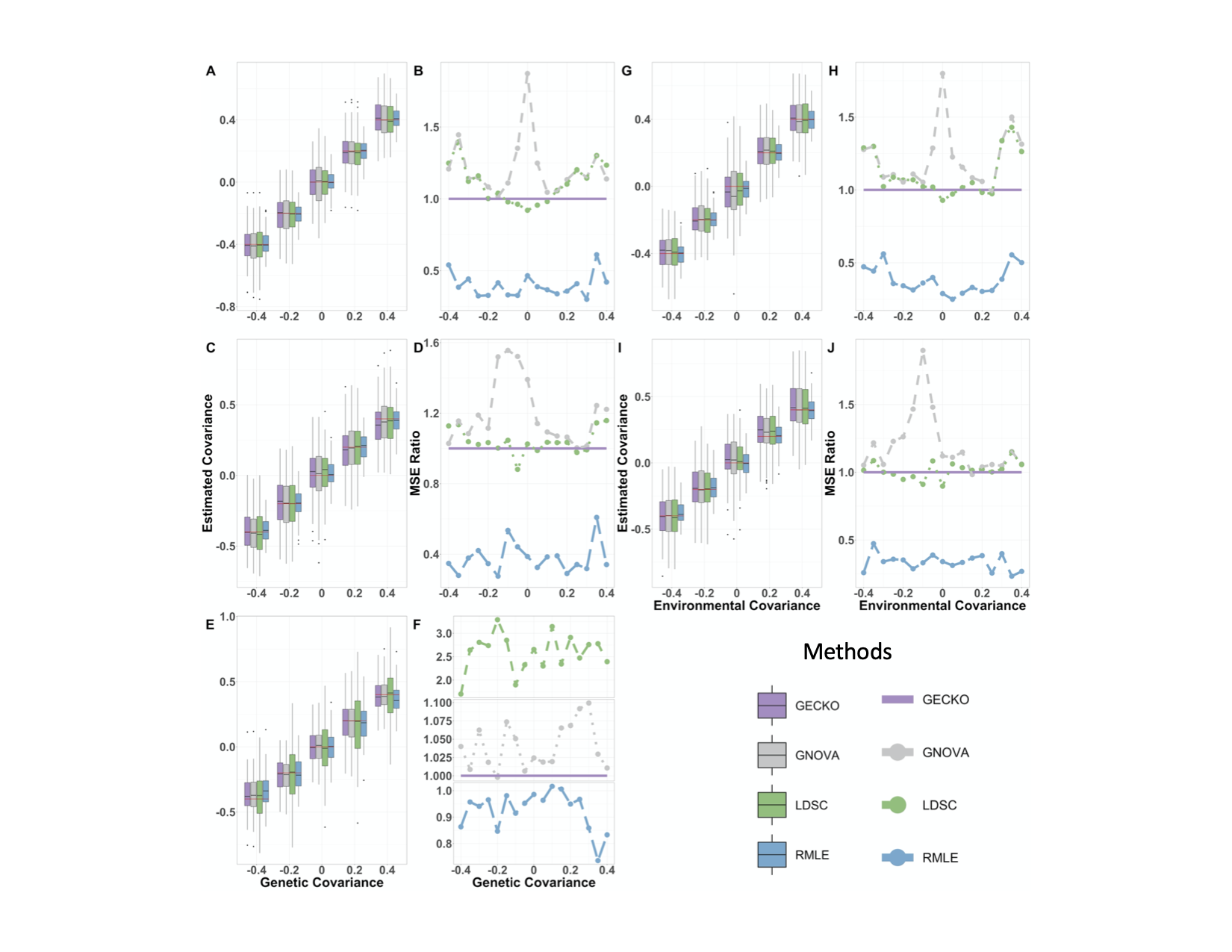

Supplement: S4 Fig — Compared methods include GECKO (purple), GNOVA (grey), LDSC (green), and mvlmm(blue). Results are shown for the one study design (first row: A, B, G, H), two partially overlapped study design (second row: C, D, I, J), and two separate study design (third row: E, F). Boxplots display estimated genetic covariances (A, C, E) and environmental covariances (G, I) on y-axis versus the true covariances on x-axis across simulation replicates. Estimation accuracy is measured by the ratio of mean square errors (MSE), which contrast the MSE from GNOVA, LDSC or mvlmm with respect to GECKO, across various true covariances on x-axis, for genetic (B, D, F) and environmental covariances (H, J). An MSE ratio below one suggests that GECKO performs worse than the other method; above one otherwise. (TIF) [file pgen.1009293.s004.tif]

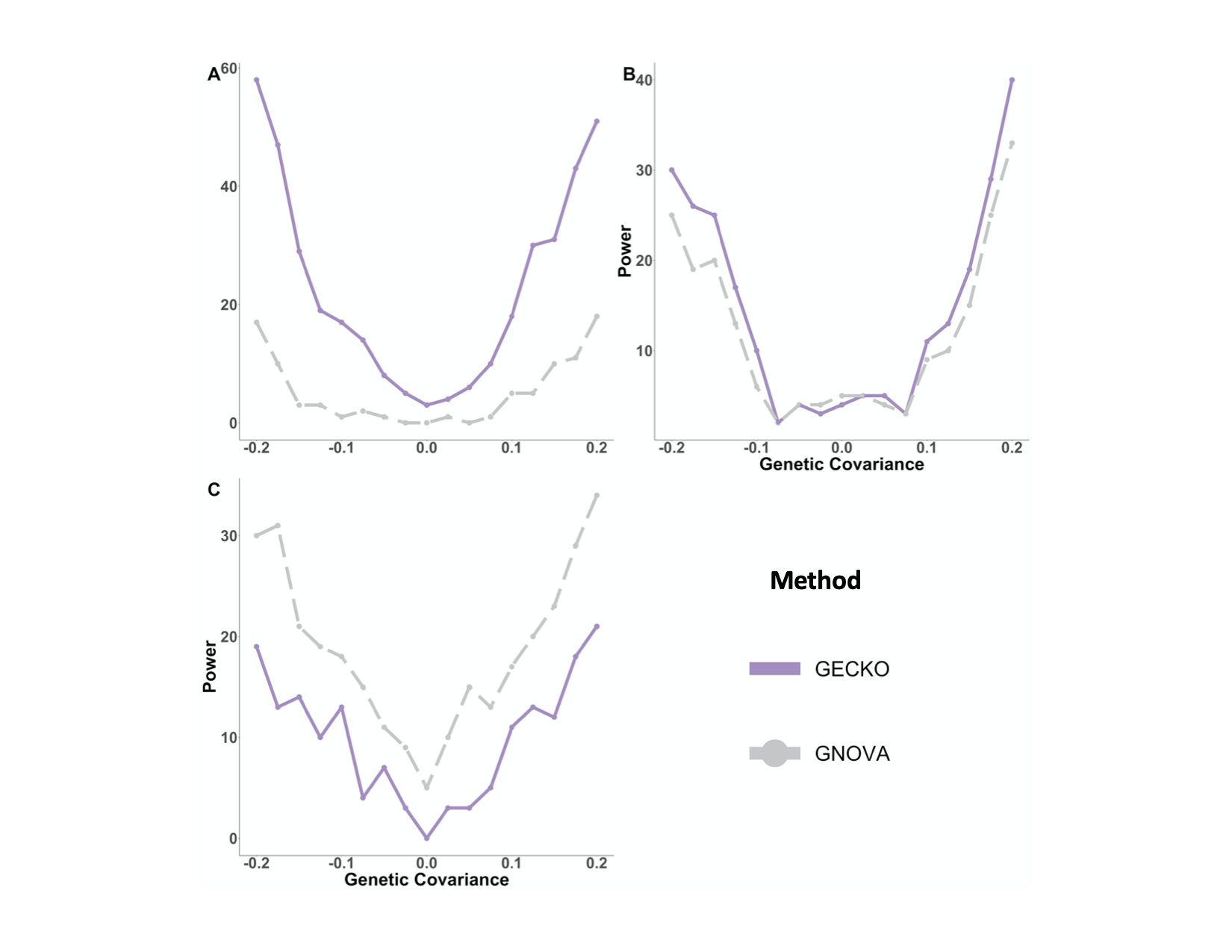

Supplement: S5 Fig — Compared methods include GECKO (purple, solid line), GNOVA (grey, dotted line).Results are shown for the one study design (first row: A, D), two partially overlapped study design (second row: B, E) and two separate study design (third row: C). Power (y-axis) of different methods are shown with respect to the true covariances (x-axis) for detecting non-zero genetic covariances (A, B, C) and environmental covariances (D, E). Power is shown based on a type I error of 0.05 but not a nominal p-value of 0.05. The power of GNOVA for detecting non-zero environmental covariance is not shown because GNOVA cannot test for environmental covariance. (TIF) [file pgen.1009293.s005.tif]

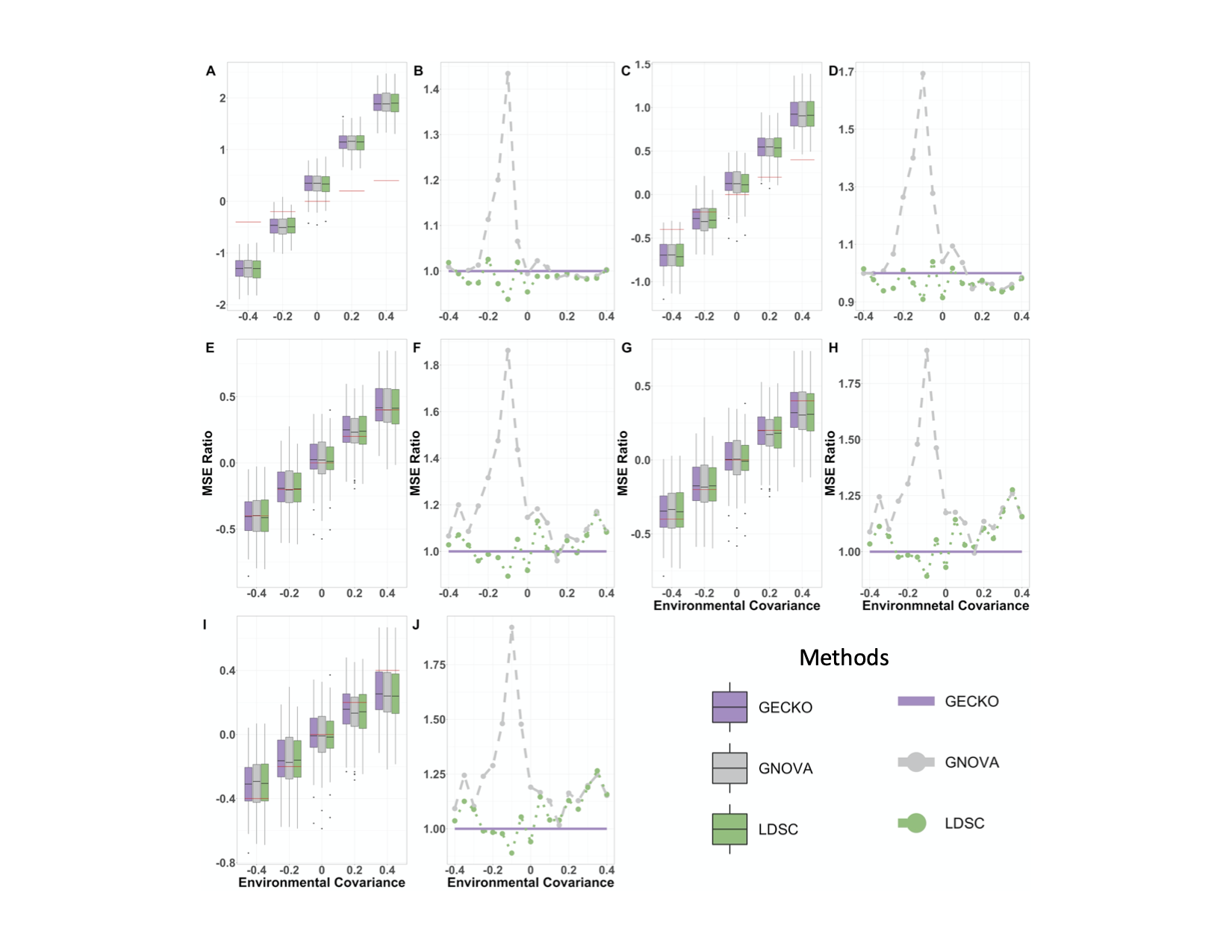

Supplement: S6 Fig — Compared methods include GECKO (purple), GNOVA (grey), and LDSC (green). Results are shown for the one study design (first row: A, B, G, H), two partially overlapped study design (second row: C, D, I, J), and two separate study design (third row: E, F). Boxplots display estimated genetic covariances (A, C, E) and environmental covariances (G, I) on y-axis versus the true covariances on x-axis across simulation replicates. Estimation accuracy is measured by the ratio of mean square errors (MSE), which contrast the MSE from GNOVA or LDSC with respect to GECKO, across various true covariances on x-axis, for genetic (B, D, F) and environmental covariances (H, J). An MSE ratio below one suggests that GECKO performs worse than the other method; above one otherwise. (TIF) [file pgen.1009293.s006.tif]

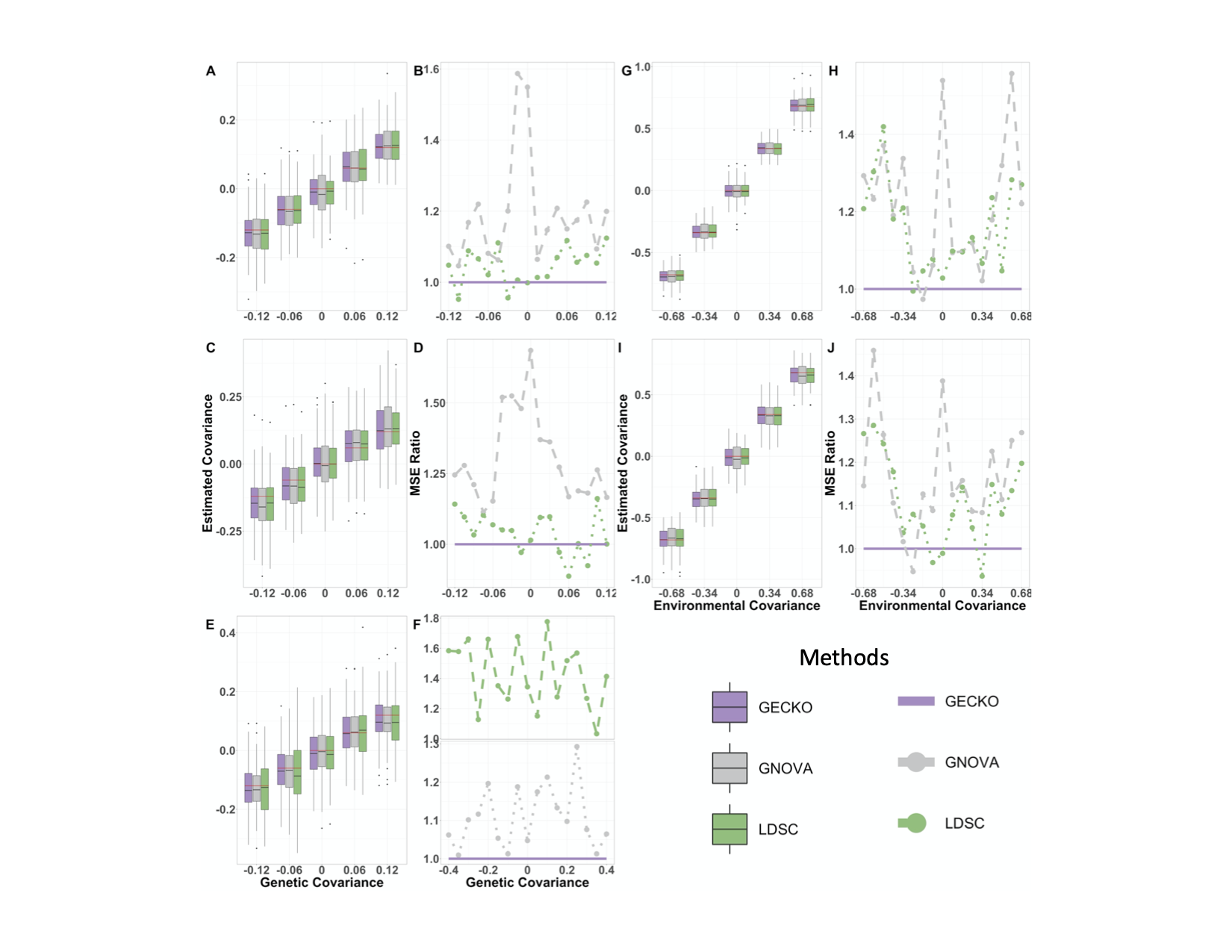

Supplement: S7 Fig — Compared methods include GECKO (purple), GNOVA (grey), and LDSC (green). Results are shown for the one study design (first row: A, B, G, H), two partially overlapped study design (second row: C, D, I, J), and two separate study design (third row: E, F). Boxplots display estimated genetic covariances (A, C, E) and environmental covariances (G, I) on y-axis versus the true covariances on x-axis across simulation replicates. Estimation accuracy is measured by the ratio of mean square errors (MSE), which contrast the MSE from GNOVA or LDSC with respect to GECKO, across various true covariances on x-axis, for genetic (B, D, F) and environmental covariances (H, J). An MSE ratio below one suggests that GECKO performs worse than the other method; above one otherwise. (TIF) [file pgen.1009293.s007.tif]

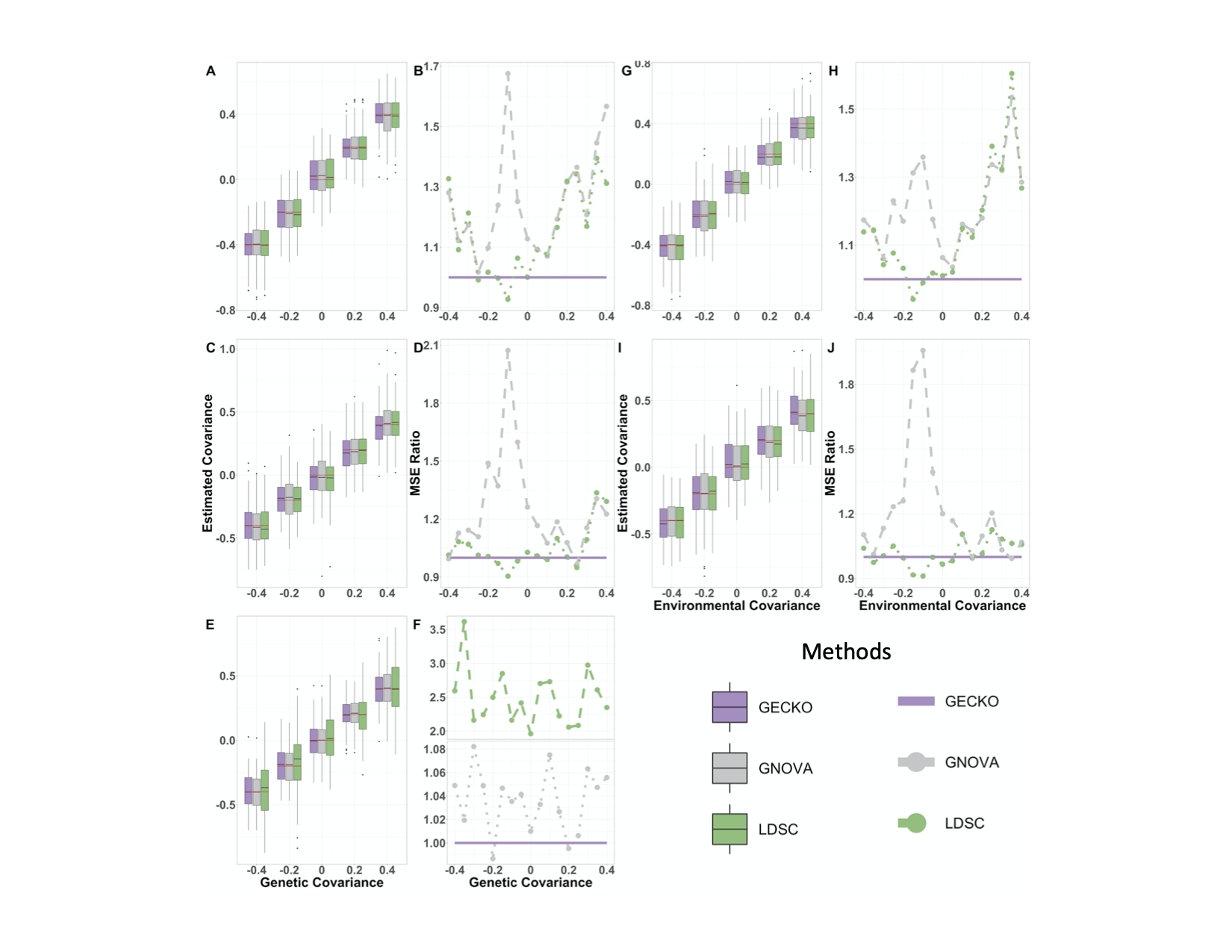

Supplement: S8 Fig — Compared methods include GECKO (purple), GNOVA (grey), and LDSC (green). Results are shown for the one study design (first row: A, B, G, H), two partially overlapped study design (second row: C, D, I, J), and two separate study design (third row: E, F). Boxplots display estimated genetic covariances (A, C, E) and environmental covariances (G, I) on y-axis versus the true covariances on x-axis across simulation replicates. Estimation accuracy is measured by the ratio of mean square errors (MSE), which contrast the MSE from GNOVA or LDSC with respect to GECKO, across various true covariances on x-axis, for genetic (B, D, F) and environmental covariances (H, J). An MSE ratio below one suggests that GECKO performs worse than the other method; above one otherwise. (TIF) [file pgen.1009293.s008.tif]

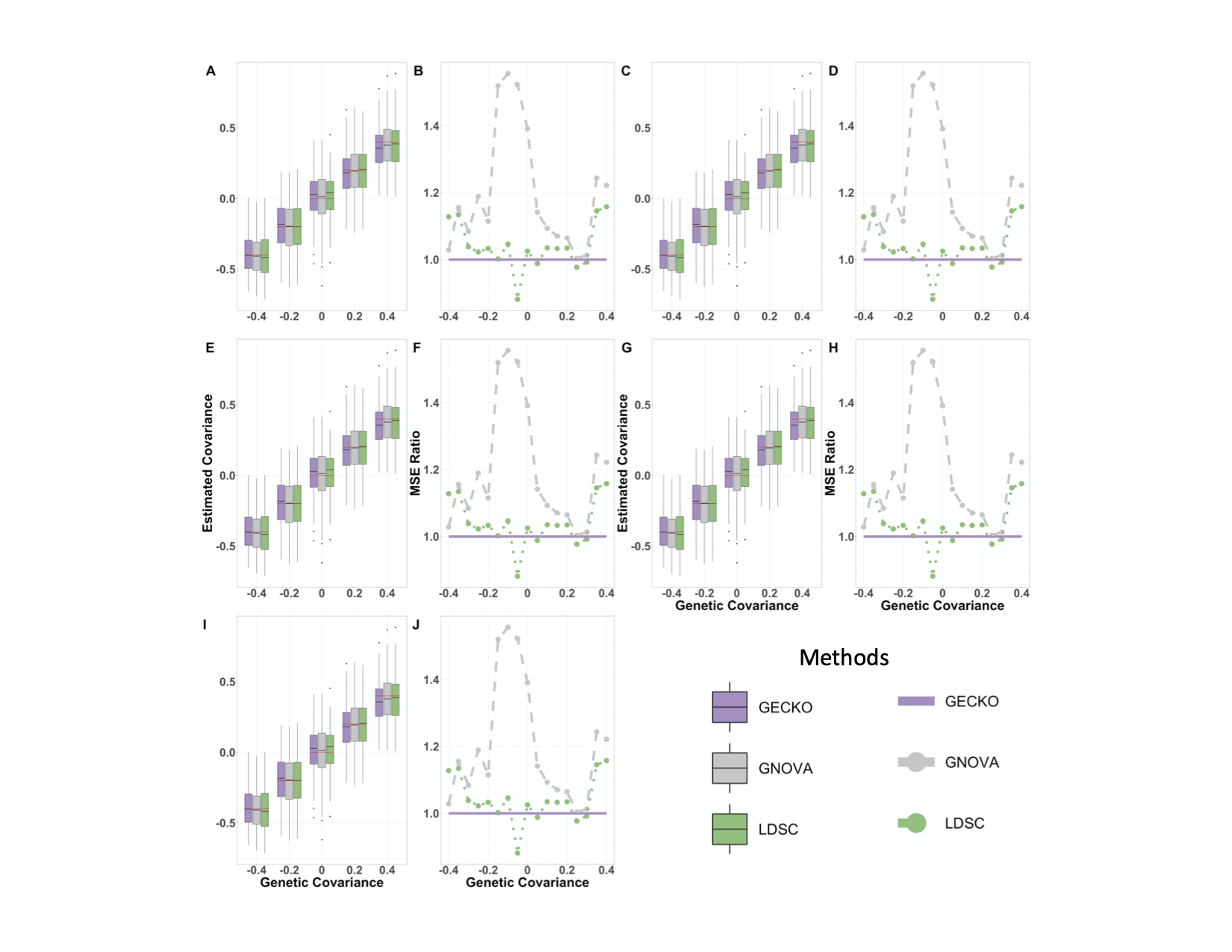

Supplement: S9 Fig — Compared methods include GECKO (purple), GNOVA (grey), and LDSC (green). Results are shown for ns being 250 (A, B), 500 (C, D), 1000 (E, F), 1250 (G, H), 1500 (I, J); Boxplots display estimated genetic covariances (A, C, E, G, I) on y-axis versus the true covariances on x-axis across simulation replicates. Estimation accuracy is measured by the ratio of mean square errors (MSE), which contrast the MSE from GNOVA or LDSC with respect to GECKO, across various true covariances on x-axis, for genetic covariances (B, D, F, H, J). An MSE ratio below one suggests that GECKO performs worse than the other method; above one otherwise. (TIF) [file pgen.1009293.s009.tif]

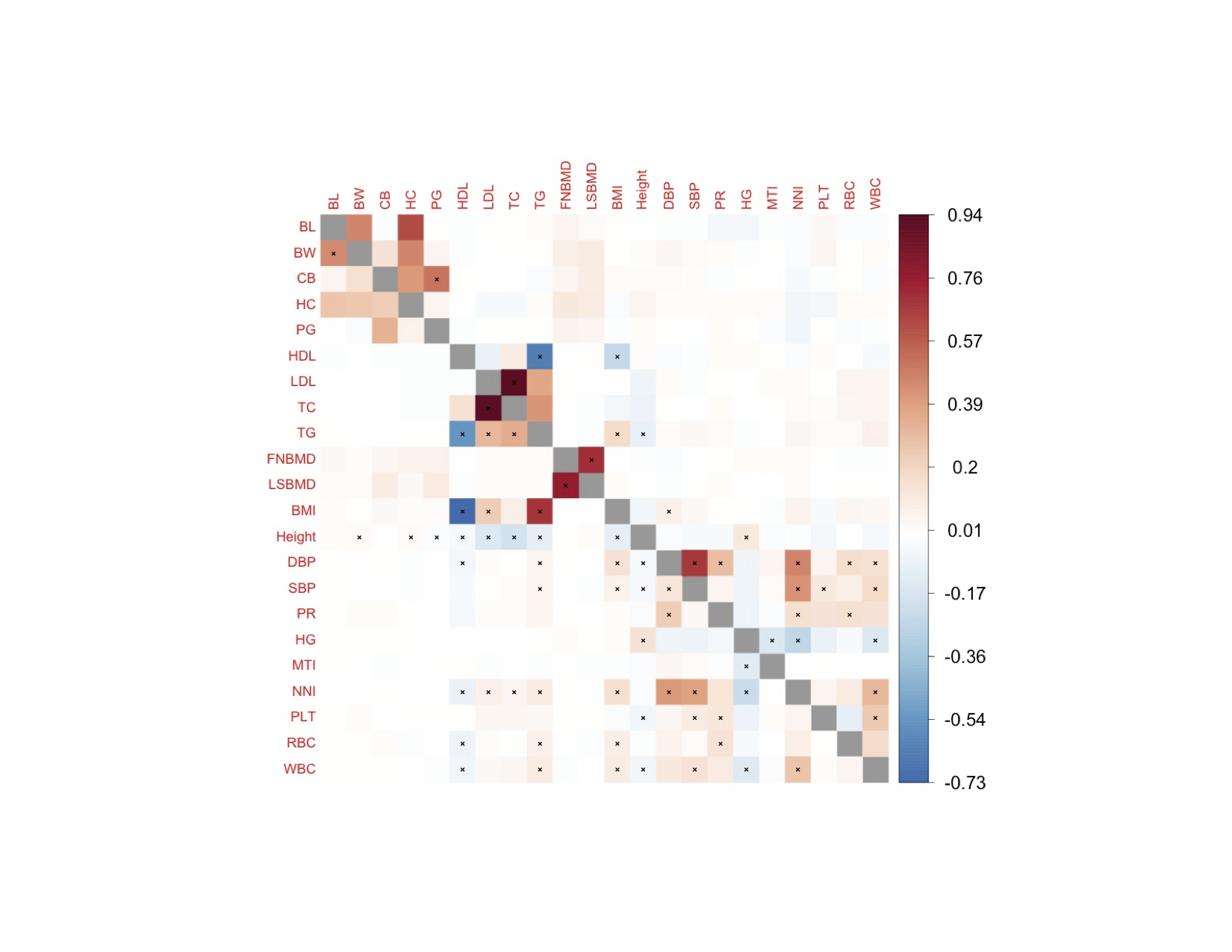

Supplement: S11 Fig — The upper triangular represents the genetic correlation estimates by LDSC while the lower triangular respresents the estimates by GECKO. The cross in the square represents the significant genetic correlation after Bonferroni Correlation. (TIF) [file pgen.1009293.s011.tif]
