# Supplementary material for: Accurate genetic and environmental covariance estimation with composite likelihood in genome-wide association studies
Source: PLoS Genet. 2021 Jan 4;17(1):e1009293. doi: 10.1371/journal.pgen.1009293 (PMC7808654; doi:10.1371/journal.pgen.1009293)
Supplement: S1 Text — (DOCX) [file pgen.1009293.s016.docx]

**Supplementary Text**

**Model Details**

**Marginal Likelihood Derivation**

The mvLMM model is described in detail in the main text. Briefly, we consider two standardized traits $y_{1}$ and $y_{2}$ collected from two studies with sample sizes $n_{1}$ and $n_{2}$_,_ respectively. We denote $n_{s}$ as the number of overlapped individuals in both studies. We denote $X_{1}$ and $X_{2}$ as the standardized genotype matrices collected on *m* SNPs in the two studies, with dimensionality being $n_{1}\times m$ and $n_{2}\times m$ respectively. We assume that $X_{1}$ and $X_{2}$are random design matrices. We further denote $X_{s}$ as the $n_{s}\times m$genotype matrix for overlapped individuals. We consider the following models

| $y_{1}=X_{1}\beta_{1}+\epsilon_{1},$  $y_{2}=X_{2}\beta_{2}+\epsilon_{2},$ | (1) |
| --- | --- |

with modeling assumptions on the effect sizes being

$$\left( \begin{matrix} \beta_{1} \\ \beta_{2} \end{matrix} \right)\sim MVN\left( \begin{matrix} \left( \begin{matrix} 0 \\ 0 \end{matrix} \right) & \frac{1}{m}\left( \begin{matrix} h_{1}^{2}I_{p*p} & \rho_{g}I_{p*p} \\ \rho_{g}I_{p*p} & h_{2}^{2}I_{p*p} \end{matrix} \right) \end{matrix} \right).$$

For the individual *i* who has phenotypes measured in both studies, we assume

$$\left( \begin{matrix} \epsilon_{1i} \\ \epsilon_{2i} \end{matrix} \right)\sim MVN\left( \begin{matrix} \left( \begin{matrix} 0 \\ 0 \end{matrix} \right) & \left( \begin{matrix} \left( 1-h_{1}^{2} \right) & \rho_{e} \\ \rho_{e} & \left( 1-h_{2}^{2} \right) \end{matrix} \right) \end{matrix} \right),$$

while for non-overlapped individuals *i* and *j*, we assume

$$\epsilon_{1i} \sim N\left( 0, 1-h_{1}^{2} \right),$$

$$\epsilon_{2j} \sim N\left( 0, 1-h_{2}^{2} \right).$$

We obtained summary statistics of the two marginal z-scores for the *j*’th SNP as $z_{j}= \left( \begin{matrix} z_{1j} \\ z_{2j} \end{matrix} \right)=\left( \begin{matrix} \frac{X_{1j}^{T}}{\sqrt{n_{1}}} \\ 0 \end{matrix}\begin{matrix} 0 \\ \frac{X_{2j}^{T}}{\sqrt{n_{2}}} \end{matrix} \right)\left( \begin{matrix} y_{1} \\ y_{2} \end{matrix} \right)$. Based on the modeling assumption in equation (1), $\left( \begin{matrix} y_{1} \\ y_{2} \end{matrix} \right)$ follow a multivariate normal distribution. Because $z_{j}$ as a linear transformation of $\left( \begin{matrix} y_{1} \\ y_{2} \end{matrix} \right)$, it also follows a multivariate normal distribution. Therefore, the joint distribution for the two z-scores of each SNP is a multivariate normal distribution, with parameters obtainable by examining their first and second moments. We examine the first and second moments of z-scores based on two different assumptions: the random design matrix assumption where we assume that the genotype matrix *X* is a random variable; and the fixed design matrix assumption where we assume that the genotype matrix *X* is fixed. We will show that the marginal distribution for the two z-scores is the same under the two different assumptions.

**Marginal Likelihood Under Random Design Matrix Assumption**

Under the random design assumption, the genotype matrix is a random quantity. We can obtain the first and second moments for the two z-scores as

$$E\left[ z_{1j} \right]=\frac{E(X_{1j}^{T}y_{1})}{\surd n_{1}}= \frac{E(X_{1j}^{T}\left( X_{1}\beta_{1}+\epsilon_{1} \right))}{\surd n_{1}}= 0,$$

$$E\left[ z_{2j} \right]=\frac{E(X_{2j}^{T}y_{2})}{\surd n_{2}}= \frac{E(X_{2j}^{T}\left( X_{2}\beta_{2}+\epsilon_{2} \right))}{\surd n_{2}}= 0,$$

| $var\left( z_{1j} \right)=E\left( z_{1j}^{2} \right)-E\left( z_{1j} \right)^{2}$ $=\frac{E\left( X_{1j}^{T}\left( X_{1}\beta_{1}+\epsilon_{1} \right)\left( X_{1}\beta_{1}+\epsilon_{1} \right)^{T}X_{1j} \right)}{n_{1}}$ $=\frac{h_{1}^{2}}{n_{1}m}E\left[ X_{1j}^{T}X_{1}X_{1}^{T}X_{1j} \right]+\frac{\left( 1-h_{1}^{2} \right)}{n_{1}}E(X_{1j}^{T}X_{1j})$ | (2) |
| --- | --- |

Similarly, we could get,

| $var\left( z_{2j} \right)=\frac{h_{2}^{2}}{n_{2}m}E\left[ X_{2j}^{T}X_{2}X_{2}^{T}X_{2j} \right]+\frac{\left( 1-h_{2}^{2} \right)}{n_{1}}E(X_{2j}^{T}X_{2j})$ | (3) |
| --- | --- |

For the covariance term we have,

| $cov\left( z_{1j},z_{2j} \right)= \frac{1}{\sqrt{n_{1}n_{2}}}E\left( X_{1j}^{T}\left( X_{1}\beta_{1}+\epsilon_{1} \right)\left( X_{2}\beta_{2}+\epsilon_{2} \right)^{T}X_{2j} \right)$ $= \frac{1}{\sqrt{n_{1}n_{2}}}E\left( X_{1j}^{T}X_{1}\beta_{1}\beta_{2}^{T}X_{2}^{T}X_{2j}+X_{1j}^{T}\epsilon_{1}\epsilon_{2}^{T}X_{2j} \right)$ $=\frac{\rho_{g}}{m\sqrt{n_{1}n_{2}}}{E(X}_{1j}^{T}X_{1}X_{2}^{T}X_{2j})+ \frac{\rho_{e}}{\sqrt{n_{1}n_{2}}}{E(X}_{sj}^{T}X_{sj}).$ | (4) |
| --- | --- |

While we have computed the covariance for the marginal z-scores in equations (2-4), these covariances are functions of the genotype matrices $X_{1}, X_{2}$ and $X_{s}$ due to random matrix design. To facilitate computation using summary statistics, we compute the expectation of the covariance by making additional assumptions on the random genotypes. Specifically, we follow MQS[1] and assume that individuals are all independent from each other and that the standardized genotypes of the *i*’th and *j*’th SNP for each individual have zero means and are correlated with each other with correlation coefficient $r_{ij}^{2}$. Further, we denote the LD score for the *j*’th SNP as $l_{j}= \sum_{i=1}^{m} r_{ji}^{2}$. Under these assumptions, we can compute the expectations as

$$E\left[ X_{1j}^{T}X_{1}X_{1}^{T}X_{1j} \right]=E\left[ \sum_{k=1}^{m} X_{1j}^{T}X_{1k}X_{1k}^{T}X_{1j} \right]$$

$$=E\left[ \sum_{k=1}^{m} (\sum_{i=1}^{n_{1}} x_{1ij}x_{1ik})(\sum_{i=1}^{n_{1}} x_{1ij}x_{1ik}) \right]$$

$$=E\left[ \sum_{k=1}^{m} \left( \sum_{i=1}^{n_{1}} x_{1ij}^{2}x_{1ik}^{2}+\sum_{i\neq i^{'}} x_{1ij}x_{1ik}x_{1i^{'}j}x_{1i^{'}k} \right) \right]$$

$$=\sum_{k=1}^{m} E\left( \sum_{i=1}^{n_{1}} x_{1ij}^{2}x_{1ik}^{2}+\sum_{i\neq i^{'}} x_{1ij}x_{1ik}x_{1i^{'}j}x_{1i^{'}k} \right),$$

where $x_{1ij}$ denotes the genotype for *i*’th individual and *j*’th SNP in the first study. By Isserlis’ theorem, we have

$$E{(x}_{1ij}x_{1ik}x_{1i^{'}j}x_{1i^{'}k})$$

$$=E{(x}_{1ij}x_{1ik})E(x_{1i^{'}j}x_{1ik})+E{(x}_{1ij}x_{1i^{'}j})E(x_{1ik}x_{1i^{'}k})+E{(x}_{1ij}x_{1i^{'}k})E(x_{1ik}x_{1i^{'}j})$$

$$=r_{jk}^{2} ,$$

$$E{(x}_{1ij}x_{1ik})=E\left( x_{1i^{'}j}x_{1i^{'}k} \right)= r_{jk},$$

$$E{(x}_{1ij}x_{1i^{'}j})=E\left( x_{1ik}x_{1i^{'}k} \right)=E{(x}_{1ij}x_{1i^{'}k})=E\left( x_{1ik}x_{1i^{'}j} \right)=0.$$

Since $i$ and $i^{'}$are referred to two different individuals, we have

$$E{(x}_{1ij}^{2}x_{1ik}^{2})={E{(x}_{1ij}x_{1ik})}^{2}+var{(x}_{1ij}x_{1ik}),$$

where

$$var{(x}_{1ij}x_{1ik})=E\left( var{(x}_{1ij}x_{1ik}\left| x_{1ik} \right) \right)+var\left( E\left( {(x}_{1ij}x_{1ik}\left| x_{1ik} \right) \right) \right).$$

Consequently,

$$E\left( var{(x}_{1ij}x_{1ik}\left| x_{1ik} \right) \right)=E\left( x_{1ik}^{2}\left( 1-r_{jk}^{2} \right) \right)=1-r_{jk}^{2},$$

$$var\left( E\left( {(x}_{1ij}x_{1ik}\left| x_{1ik} \right) \right) \right)=var\left( x_{1ik}^{2}r_{jk} \right)=2r_{jk}^{2},$$

$$E{(x}_{1ij}^{2}x_{1ik}^{2})={E{(x}_{1ij}x_{1ik})}^{2}+var{(x}_{1ij}x_{1ik})= 1+2r_{jk}^{2},$$

| $E\left[ X_{1j}^{T}X_{1}X_{1}^{T}X_{1j} \right]=\sum_{k=1}^{m} E\left( \sum_{i=1}^{n_{1}} x_{1ij}^{2}x_{1ik}^{2}+\sum_{i\neq i^{'}} x_{1ij}x_{1ik}x_{1i^{'}j}x_{1i^{'}k} \right)$ $=\sum_{k=1}^{m} \left( n_{1}\left( 1+{2r}_{jk}^{2} \right)+n_{1}\left( n_{1}-1 \right)r_{jk}^{2} \right)$ $\approx(\sum_{k=1}^{m} n_{1}^{2}r_{jk}^{2})+n_{1}m.$ | (5) |
| --- | --- |

Similarly, we have

| $E\left[ X_{2j}^{T}X_{2}X_{2}^{T}X_{2j} \right]\approx(\sum_{k=1}^{m} n_{2}^{2}r_{jk}^{2})+n_{2}m.$ | (6) |
| --- | --- |

For the covariance term, we denote $X_{1u}$ as the $\left( n_{1}-n_{s} \right)$ by *m* genotype matrix for the unique individuals in the first study and $X_{2u}$ as the $\left( n_{2}-n_{s} \right)$ by *m* genotype matrix for the unique individuals in the first study. Therefore, we have $X_{1}=\left( X_{s}, X_{1u} \right)$and $X_{2}=\left( X_{s}, X_{2u} \right)$. Consequently, we have

| $E\left[ X_{1j}^{T}X_{1}X_{2}^{T}X_{2j} \right]=E\left[ \left( X_{sj}, X_{1uj} \right)^{T}\left( X_{s}, X_{1u} \right) \left( X_{s},X_{2u} \right)^{T}\left( X_{sj},X_{2uj} \right) \right]$  $= E\left[ X_{sj}^{T}X_{s}X_{s}^{T}X_{sj}+X_{1uj}^{T}X_{1u}X_{2u}^{T}X_{2uj}+X_{1uj}^{T}X_{1u}X_{s}^{T}X_{sj}+X_{sj}^{T}X_{s}X_{2u}^{T}X_{2uj} \right]$ $=E\left[ \sum_{k=1}^{m} X_{sj}^{T}X_{sk}X_{sk}^{T}X_{sj}+X_{1uj}^{T}X_{1uk}X_{2uk}^{T}X_{2uj}+X_{1uj}^{T}X_{1uk}X_{sk}^{T}X_{sj}+X_{sj}^{T}X_{sk}X_{2uk}^{T}X_{2uj} \right].$ | (7) |
| --- | --- |

$.$From the above equations (5) and (6), we know that

| $E\left[ \sum_{k=1}^{m} X_{nsj}^{T}X_{nsk}X_{nsk}^{T}X_{nsj} \right] \approx(\sum_{k=1}^{m} n_{s}^{2}r_{jk}^{2})+n_{s}m.$ | (8) |
| --- | --- |

For the remaining terms in equation (7), we have

| $E\left[ \sum_{k=1}^{m} X_{1sj}^{T}X_{1sk}X_{2sk}^{T}X_{2sj} \right]=E\left[ \sum_{k=1}^{m} \{(\sum_{i=1}^{n_{1s}} x_{1sij}x_{1sik})*(\sum_{i^{'}=1}^{n_{2s}} x_{2sij}x_{2sik})\} \right]$ $=E\left[ \sum_{k=1}^{m} \sum_{i,i^{'}} x_{1sij}x_{1sik}x_{2sij}x_{2sik} \right]$ $=\sum_{k=1}^{m} {(n}_{1}-n_{s}){(n}_{2}-n_{s}) r_{jk}^{2},$ | (9) |
| --- | --- |

and

$$E\left[ x_{1sij}x_{1sik}x_{2sij}x_{2sik} \right]$$

$$=E\left[ x_{1sij}x_{1sik} \right]E\left[ x_{2sij}x_{2sik} \right]+E\left[ x_{1sij}x_{2sij} \right]E\left[ x_{1sik}x_{2sik} \right]+E\left[ x_{1sij}x_{2sik} \right]E\left[ x_{1sik}x_{2sij} \right]$$

$$=E\left[ x_{1sij}x_{1sik} \right]E\left[ x_{2sij}x_{2sik} \right]$$

$$={(n}_{1}-n_{s}){(n}_{2}-n_{s}) r_{jk}^{2},$$

and

| $E\left[ \sum_{k=1}^{m} X_{1sj}^{T}X_{1sk}X_{nsk}^{T}X_{nsj} \right]=\sum_{k=1}^{m} {(n}_{1}-n_{s})n_{s} r_{jk}^{2},$ | (10) |
| --- | --- |
| $E\left[ \sum_{k=1}^{m} X_{2sj}^{T}X_{2sk}X_{nsk}^{T}X_{nsj} \right]=\sum_{k=1}^{m} {(n}_{2}-n_{s})n_{s} r_{jk}^{2}.$ | (11) |

Combining equations (8–11), we have

$$E\left[ X_{1j}^{T}X_{1}X_{2}^{T}X_{2j} \right]=\left( \sum_{k=1}^{m} n_{1}n_{2}r_{jk}^{2} \right)+n_{s}m.$$

Therefore, we can approximate the covariance matrix for $\left( \begin{matrix} z_{1j} \\ z_{2j} \end{matrix} \right)$ using its expectation under random genotypes as

$$cov\left( \left( \begin{matrix} z_{1j} \\ z_{2j} \end{matrix} \right) \right)=\left( \begin{matrix} h_{1}^{2}\left( \frac{l_{j}n_{1}}{m}+1 \right) & \rho_{g}\left( \frac{l_{j}\sqrt{n_{1}n_{2}}}{m}+\frac{n_{s}}{\sqrt{n_{1}n_{2}}} \right) \\ \rho_{g}\left( \frac{l_{j}\sqrt{n_{1}n_{2}}}{m}+\frac{n_{s}}{\sqrt{n_{1}n_{2}}} \right) & h_{2}^{2}\left( \frac{l_{j}N_{2}}{m}+1 \right) \end{matrix} \right)+\left( \begin{matrix} \left( 1-h_{1}^{2} \right) & \frac{\rho_{e}n_{s}}{\sqrt{n_{1}n_{2}}} \\ \frac{\rho_{e}n_{s}}{\sqrt{n_{1}n_{2}}} & \left( 1-h_{2}^{2} \right) \end{matrix} \right).$$

Therefore, the joint distribution of the two marginal z-scores for the *j*’th SNP is

| $\left( \begin{matrix} z_{1j} \\ z_{2j} \end{matrix} \right) \sim MVN\left( \begin{matrix} \left( \begin{matrix} 0 \\ 0 \end{matrix} \right) & \frac{l_{j}}{m}\left( \begin{matrix} h_{1}^{2}n_{1} & \rho_{g}\sqrt{n_{1}n_{2}} \\ \rho_{g}\sqrt{n_{1}n_{2}} & h_{2}^{2}n_{2} \end{matrix} \right) +\left( \begin{matrix} 1 & \frac{\rho n_{s}}{\sqrt{n_{1}n_{2}}} \\ \frac{\rho n_{s}}{\sqrt{n_{1}n_{2}}} & 1 \end{matrix} \right) \end{matrix} \right).$ | (12) |
| --- | --- |

**Marginal Likelihood Under Fixed Design Matrix Assumption**

Above, we have obtained the marginal distribution for the two z-scores under random design matrix assumption. Here, we derive the same marginal distribution for the two z-scores under random design matrix assumption where the genotype matrix *X* is assumed to be fixed. Specifically, we have

| $cov\left( z_{1j},z_{2j} \right)= \frac{1}{\sqrt{n_{1}n_{2}}}E\left( X_{1j}^{T}\left( X_{1}\beta_{1}+\epsilon_{1} \right)\left( X_{2}\beta_{2}+\epsilon_{2} \right)^{T}X_{2j} \right)$ $= \frac{1}{\sqrt{n_{1}n_{2}}}E\left( X_{1j}^{T}X_{1}\beta_{1}\beta_{2}^{T}X_{2}^{T}X_{2j}+X_{1j}^{T}\epsilon_{1}\epsilon_{2}^{T}X_{2j} \right)$ $=\frac{\rho_{g}}{m\sqrt{n_{1}n_{2}}}{(X}_{1j}^{T}X_{1}X_{2}^{T}X_{2j})+ \frac{\rho_{e}n_{s}}{\sqrt{n_{1}n_{2}}},$ | (13) |
| --- | --- |

where the first term is

| $X_{1j}^{T}X_{1}X_{2}^{T}X_{2j}=\left( X_{sj}, X_{1uj} \right)^{T}\left( X_{s}, X_{1u} \right) \left( X_{s},X_{2u} \right)^{T}\left( X_{sj},X_{2uj} \right)= X_{sj}^{T}X_{s}X_{s}^{T}X_{sj}+X_{1uj}^{T}X_{1u}X_{2u}^{T}X_{2uj}+X_{1uj}^{T}X_{1u}X_{s}^{T}X_{sj}+X_{sj}^{T}X_{s}X_{2u}^{T}X_{2uj}= \sum_{k=1}^{m} X_{sj}^{T}X_{sk}X_{sk}^{T}X_{sj}+X_{1uj}^{T}X_{1uk}X_{2uk}^{T}X_{2uj}+X_{1uj}^{T}X_{1uk}X_{sk}^{T}X_{sj}+X_{sj}^{T}X_{sk}X_{2uk}^{T}X_{2uj}.$ | (14) |
| --- | --- |

To compute the above term, we have

| $X_{sj}^{T}X_{sk}X_{sk}^{T}X_{sj}= \sum_{k=1}^{m} X_{sj}^{T}X_{sk}X_{sk}^{T}X_{sj}$ $= \sum_{k=1}^{m} (\sum_{i=1}^{n_{s}} x_{sij}x_{sik})(\sum_{i=1}^{n_{s}} x_{sij}x_{sik}))$ $= \sum_{k=1}^{m} \left( n_{s}*\tilde{r_{sjk}} \right)\left( n_{s}*\tilde{r_{sjk}} \right)$ $=\sum_{k=1}^{m} n_{s}^{2} \tilde{r}_{sjk}^{2}.$ | (15) |
| --- | --- |

Similarly, we have

$$X_{1uj}^{T}X_{1uk}X_{2uk}^{T}X_{2uj}= \sum_{k=1}^{m} (n_{1}-n_{s})(n_{2}-n_{s})\tilde{r}_{1jk}* \tilde{r}_{2jk},$$

$$X_{1uj}^{T}X_{1uk}X_{sk}^{T}X_{sj}=\sum_{k=1}^{m} (n_{1}-n_{s})n_{s}\tilde{r}_{1jk}* \tilde{r}_{sjk},$$

$$X_{sj}^{T}X_{sk}X_{2uk}^{T}X_{2uj}= \sum_{k=1}^{m} n_{s}(n_{2}-n_{s})\tilde{r}_{sjk}* \tilde{r}_{2jk},$$

$$X_{1j}^{T}X_{1}X_{2}^{T}X_{2j}= \sum_{k=1}^{m} n_{s}^{2} \tilde{r}_{sjk}^{2}+\left( n_{1}-n_{s} \right)\left( n_{2}-n_{s} \right)\tilde{r}_{1ujk}* \tilde{r}_{2ujk}+\left( n_{1}-n_{s} \right)n_{s}\tilde{r}_{1ujk}* \tilde{r}_{sjk}+n_{s}\left( n_{2}-n_{s} \right)\tilde{r}_{sjk}* \tilde{r}_{2ujk}.$$

Above, $\tilde{r}_{sjk}^{2}$ is the sample *R^2^* obtained in the GWAS data. Because we often do not have individual-level genotype data in the GWAS, we aim to use the SNP correlation ($r_{jk})$ computed in a reference panel to approximate the sample correlations $\tilde{r}_{1ujk}, \tilde{r}_{2ujk}, \tilde{r}_{sjk}$. Specifically, because $E\left[ \tilde{r}_{sjk}^{2} \right]= r_{jk}^{2}+\frac{1}{n_{s}}$, we have

$$X_{1j}^{T}X_{1}X_{2}^{T}X_{2j}= \sum_{k=1}^{m} n_{s}^{2} \tilde{r}_{sjk}^{2}+\left( n_{1}-n_{s} \right)\left( n_{2}-n_{s} \right)\tilde{r}_{1ujk}* \tilde{r}_{2ujk}+\left( n_{1}-n_{s} \right)n_{s}\tilde{r}_{1ujk}* \tilde{r}_{sjk}+n_{s}\left( n_{2}-n_{s} \right)\tilde{r}_{sjk}* \tilde{r}_{2ujk}\approx\left( \sum_{k=1}^{m} n_{1}n_{2}r_{jk}^{2} \right)+n_{s}m.$$

Thus, the covariance for the two z-scores is

| $cov\left( z_{1j},z_{2j} \right)=\frac{\rho_{g}}{m\sqrt{n_{1}n_{2}}}{(X}_{1j}^{T}X_{1}X_{2}^{T}X_{2j})+ \frac{\rho_{e}n_{s}}{\sqrt{n_{1}n_{2}}}=\rho_{g}\left( \frac{l_{j}\sqrt{n_{1}n_{2}}}{m} \right)+ \frac{\rho n_{s}}{\sqrt{n_{1}n_{2}}}.$ | (16) |
| --- | --- |

The covariance and the subsequent marginal distribution for the two z-scores derived based on fixed design matrix assumption is the same as that obtained from the random design matrix assumption. Therefore, the environmental covariance estimation of GECKO is robust to the assumption of the design matrix.

**Connection to LDSC and MQS**

Our method is connected to LDSC and the weighted version of MQS in the univariate linear mixed model case. Specifically, the univariate LDSC method without the intercept and the weighted version of MQS are both based on the following iterative algorithm:

$$h= \frac{m}{n}*\frac{\sum_{j} {(z}_{j}^{2}-1)\left( \frac{nhl_{j}}{m}+1 \right)^{-2}}{\sum_{j} {l_{j}\left( \frac{nhl_{j}}{m}+1 \right)}^{-2}},$$

where the *t*’th iteration estimate  $\hat{h}^{(t)}$ is plugged in on the right-hand side of the above equation to obtain the *(t+1)*’th iteration estimate $\hat{h}^{(t+1)}$ on the left hand side. Denoting $s_{j}=nl_{j}/m$, we notice that the above LDSC equation can be viewed as obtained by setting the derivative of the following function to zero:

$$l= -\sum_{j} \left( \frac{z_{j}^{2}}{{(s}_{j}h+1)s_{j}}-\frac{1}{s_{j}}\log\left( s_{j}h+1 \right) \right).$$

The above function is a weighted composite likelihood based on the marginal z-score $z_{j} \sim N(0,s_{j}h+1)$, with weight being 1/$s_{j}$. Therefore, in the univariate case, our method is mathematically equivalent to univariate LDSC without the intercept when we set the weight as 1/$s_{j}$, which is in proportional to the weighting choice we use 1/$l_{j}$.

**Extension to Incorporating Functional Annotations**

We extend our method to deal with SNP functional annotations. Here, we assume that SNPs are categorized into *K* different annotation categories. We denote $X_{1k}$ as the *k*’th category standardized genotype matrix with dimension $n_{1}$ by $m_{k}$, where $m_{k}$ is the number of the SNPs in the *k*’th annotation category. We consider the following extended models for each of the two traits

| $y_{1}=\sum_{k=1}^{K} X_{1k}\beta_{1k}+\epsilon_{1},$  $y_{2}=\sum_{k=1}^{K} X_{2k}\beta_{2k}+\epsilon_{2},$ | (17) |
| --- | --- |

where $\beta_{1k},\beta_{2k}$ are $m_{k}$ vectors of genotype effect sizes for SNPs in the *k*’th category; and $\epsilon_{1},\epsilon_{2}$ are vectors of environmental residual errors with sizes $n_{1}$and $n_{2}$, respectively.

We follow the standard mvLMM assumption and further assume that the *a priori* genetic effect sizes of *j*’th SNP in *k*’th annotation category on the two traits follow

| $\left( \begin{matrix} \beta_{1kj} \\ \beta_{2kj} \end{matrix} \right)\sim MVN\left( \begin{matrix} \left( \begin{matrix} 0 \\ 0 \end{matrix} \right), & \frac{1}{m_{k}}\left( \begin{matrix} h_{1k}^{2} & \rho_{gk} \\ \rho_{gk} & h_{2k}^{2} \end{matrix} \right) \end{matrix} \right),$ | (18) |
| --- | --- |

where $h_{1k}^{2}$ represents the SNP heritability of the *k*’th annotation category for the first trait; $h_{2k}^{2}$ represents the SNP heritability of the *k*’th annotation category for the second trait; and $\rho_{gk}$ represents the genetic covariance within *k*’th annotation category, which characterizes the trait covariance explained by shared genetic effects of SNPs in the *k*’th annotation category.

With the above modeling assumption, we can obtain the joint distribution for the two marginal z-scores of the *j*’th SNP as $\left( \begin{matrix} z_{1j} \\ z_{2j} \end{matrix} \right) \sim MVN\left( \begin{matrix} \left( \begin{matrix} 0 \\ 0 \end{matrix} \right) & \sum_{k=1}^{K} \frac{1}{m_{k}}\left( \begin{matrix} h_{1k}^{2}\frac{X_{1j}^{T}X_{1k}X_{1k}^{T}X_{1j}}{n_{1}} & \rho_{g}\frac{X_{1j}^{T}X_{1k}X_{2k}^{T}X_{2j}}{\sqrt{n_{1}n_{2}}} \\ \rho_{g}\frac{X_{2j}^{T}X_{2k}X_{1k}^{T}X_{1j}}{\sqrt{n_{1}n_{2}}} & h_{2k}^{2}\frac{X_{2j}^{T}X_{2k}X_{2k}^{T}X_{2j}}{n_{2}} \end{matrix} \right)+\left( \begin{matrix} \left( 1-\sum_{k=1}^{K} h_{1k}^{2} \right)\frac{X_{1j}^{T}X_{1j}}{n_{1}} & \frac{\rho_{e}X_{1j}^{T}IX_{2j}}{\sqrt{n_{1}n_{2}}} \\ \frac{\rho_{e}X_{2j}^{T}{IX}_{1j}}{\sqrt{n_{1}n_{2}}} & \left( 1-\sum_{k=1}^{K} h_{2k}^{2} \right)\frac{X_{2j}^{T}X_{2j}}{n_{2}} \end{matrix} \right) \end{matrix} \right),$(19)

Following similar strategies detailed in the first section, we can approximate the joint distribution as

| $\left( \begin{matrix} z_{1j} \\ z_{2j} \end{matrix} \right) \sim MVN\left( \begin{matrix} \left( \begin{matrix} 0 \\ 0 \end{matrix} \right) & \sum_{k=1}^{K} \frac{l_{kj}}{m_{k}}\left( \begin{matrix} h_{1k}^{2}n_{1} & \rho_{gk}\sqrt{n_{1}n_{2}} \\ \rho_{gk}\sqrt{n_{1}n_{2}} & h_{2k}^{2}n_{2} \end{matrix} \right)+\left( \begin{matrix} 1 & \frac{\rho n_{s}}{\sqrt{n_{1}n_{2}}} \\ \frac{\rho n_{s}}{\sqrt{n_{1}n_{2}}} & 1 \end{matrix} \right) \end{matrix} \right).$ | (20) |
| --- | --- |

where $\rho= \rho_{e} + {\sum_{k=1}^{K} \rho}_{gk}$. Above, the LD score for the $j_{th}$ SNP within the$k_{th}$ annotation category is denoted as$l_{kj}=\sum_{i=1}^{m_{k}} r_{ij}^{2}$, where $r_{ij}$ is the correlation coefficient between *j*’th SNP and *i*’th SNP within *k*’th annotation region, and *n* is the number of individuals in the reference panel.

**Extension for Overlapping Functional Annotations**

Here, we extend our method to deal with multiple, potentially overlapping functional annotations, based on the notations provided in the previous subsection. To do so, we consider the same model in equation (1) and rewrite the model in terms of individuals SNPs as

| $y_{1}= \sum_{q=1}^{m} x_{1q}\beta_{1q}+\epsilon_{1},$  $y_{2}= \sum_{q=1}^{m} x_{2q}\beta_{2q}+\epsilon_{2}.$ | (21) |
| --- | --- |

When q’th SNP belongs to the k’th functional annotation category, as in the previous subsection, we assume that

| $\left( \begin{matrix} \beta_{1q} \\ \beta_{2q} \end{matrix} \right)\sim MVN\left( \begin{matrix} \left( \begin{matrix} 0 \\ 0 \end{matrix} \right) & \frac{1}{m_{k}}\left( \begin{matrix} h_{1k}^{2} & \rho_{gk} \\ \rho_{gk} & h_{2k}^{2} \end{matrix} \right) \end{matrix} \right).$ | (22) |
| --- | --- |

When q’th SNP belongs to multiple functional annotation categories, we assume that

| $\left( \begin{matrix} \beta_{1q} \\ \beta_{2q} \end{matrix} \right)\sim MVN\left( \begin{matrix} \left( \begin{matrix} 0 \\ 0 \end{matrix} \right) & \sum_{q\in K} \frac{1}{m_{k}}\left( \begin{matrix} h_{1k}^{2} & \rho_{gk} \\ \rho_{gk} & h_{2k}^{2} \end{matrix} \right) \end{matrix} \right).$ | (23) |
| --- | --- |

Under the above multiple functional annotation assumption, we have

| $E\left( z_{1j}^{2} \right)=\frac{E\left( x_{1j}^{T}y_{1}y_{1}^{T}x_{1j}^{T} \right)}{n_{1}}$ $=\frac{E\left[ x_{1j}^{T}\left( \sum_{q=1}^{m} x_{1q}\beta_{1q}+\epsilon_{1} \right)\left( \sum_{q=1}^{m} x_{1q}\beta_{1q}+\epsilon_{1} \right)^{T}x_{1j} \right]}{n_{1}}$ $=\frac{E\left[ \sum_{q=1}^{m} x_{1j}^{T}x_{1q}\sum_{q\in k} \frac{h_{1k}^{2}}{m_{k}}x_{1q}^{T}x_{1j} \right]}{n_{1}}+\frac{E\left[ x_{1j}^{T}\epsilon_{1}\epsilon_{1}^{T}x_{1j} \right]}{n_{1}}$ $= E\left[ \sum_{k} \frac{h_{1k}^{2}}{m_{k}}\sum_{q\in k} x_{1j}^{T}x_{1q}x_{1q}^{T}x_{1j} \right]+\sigma_{e_{1}}^{2}.$ | (24) |
| --- | --- |

Since $\left( \begin{matrix} \beta_{1q} \\ \beta_{2q} \end{matrix} \right)$ is independent of $\left( \begin{matrix} \beta_{1j} \\ \beta_{2j} \end{matrix} \right)$ for $j\neq q$, we have

| $E{[x}_{1j}^{T}x_{1q}x_{1q}^{T}x_{1j}]\approx n_{1}^{2}r_{jq}^{2}+n_{1},$ | (25) |
| --- | --- |

as we have shown in equation (5). Consequently, we have

| $E\left[ \sum_{k} \frac{h_{1k}^{2}}{m_{k}}\sum_{q\in k} x_{1j}^{T}x_{1q}x_{1q}^{T}x_{1j} \right]=\sum_{k} \frac{h_{1k}^{2}}{m_{k}}(\sum_{q\in k} n_{1}^{2}r_{jq}^{2}+n_{1})=n_{1}^{2}\sum_{k} \frac{h_{1k}^{2}}{m_{k}}l_{kj}+ \sum_{k} \sum_{q\in k} \frac{h_{1k}^{2}}{m_{k}}n_{1}= n_{1}^{2}\sum_{k} \frac{h_{1k}^{2}}{m_{k}}l_{kj}+\sum_{q=1}^{m} \sum_{q\in k} \frac{h_{1k}^{2}}{m_{k}}n_{1},$ | (26) |
| --- | --- |

$$\sum_{q=1}^{m} \sum_{q\in k} \frac{h_{1k}^{2}}{m_{k}}n_{1}=n_{1}\sum_{q} var(\beta_{q}).$$

Therefore,

| $E\left( z_{1j}^{2} \right)=\frac{E\left( x_{1j}^{T}y_{1}y_{1}^{T}x_{1j}^{T} \right)}{n_{1}}= n_{1}\sum_{k} \frac{h_{1k}^{2}}{m_{k}}l_{kj}+\sum_{q} var(\beta_{q})+ \sigma_{e_{1}}^{2},$ | (27) |
| --- | --- |

Similarly, we could have

| $E\left( z_{2j}^{2} \right)=\frac{E\left( x_{2j}^{T}y_{2}y_{2}^{T}x_{2j}^{T} \right)}{n_{2}}= n_{2}\sum_{k} \frac{h_{2k}^{2}}{m_{k}}l_{kj}+\sum_{q} var(\beta_{q})+ \sigma_{e_{1}}^{2},$ | (28) |
| --- | --- |

For the covariance of $z_{1j}$ and $z_{2j}$, we have

| $E\left[ z_{1j}z_{2j} \right]=\frac{E\left[ x_{1j}^{T}\left( \sum_{q=1}^{m} x_{1q}\beta_{1q}+\epsilon_{1} \right)\left( \sum_{q=1}^{m} x_{2q}\beta_{2q}+\epsilon_{2} \right)^{T}x_{2j} \right]}{\sqrt{n_{1}n_{2}}}=\frac{E\left[ \sum_{q=1}^{m} x_{1j}^{T}x_{1q}\beta_{1q}\beta_{2q}^{T}x_{2q}x_{1j}^{T}+x_{1j}^{T}\epsilon_{1}\epsilon_{2}^{T}x_{2j} \right]}{\sqrt{n_{1}n_{2}}} ,$ | (29) |
| --- | --- |

| $E\left[ \sum_{q=1}^{m} x_{1j}^{T}x_{1q}\beta_{1q}\beta_{2q}^{T}x_{2q}x_{1j}^{T} \right]= E\left[ \sum_{q=1}^{m} x_{1j}^{T}x_{1q}\sum_{\left\{ q \right\}} \frac{\rho_{gk}}{m_{k}}x_{2q}x_{1j}^{T} \right]=E\left[ \sum_{q=1}^{m} x_{1j}^{T}x_{1q}\sum_{q\in k} \frac{\rho_{gk}}{m_{k}}x_{2q}x_{1j}^{T} \right]=E[\sum_{k} \frac{\rho_{gk}}{m_{k}}\sum_{q\in K} x_{1j}^{T}x_{1q}x_{2q}x_{1j}^{T}]$  $,$ | (30) |
| --- | --- |

as we have shown in equations (8-11),

| $E\left( \sum_{q\in K} x_{1j}^{T}x_{1q}x_{2q}x_{1j}^{T} \right) \approx(\sum_{q\in K} \frac{n_{1}n_{2}\rho_{gk}}{m_{k}}r_{jk}^{2})+n_{s}m_{k},$ | (31) |
| --- | --- |

$$E\left[ x_{1j}^{T}\epsilon_{1}\epsilon_{2}^{T}x_{2j} \right]=n_{s}\rho_{e}.$$

Therefore,

| $E\left[ z_{1j}z_{2j} \right]=\frac{E\left[ x_{1j}^{T}\left( \sum_{q=1}^{m} x_{1q}\beta_{1q}+\epsilon_{1} \right)\left( \sum_{q=1}^{m} x_{2q}\beta_{2q}+\epsilon_{2} \right)^{T}x_{2j} \right]}{\surd n_{1}n_{2}}=\sqrt{n_{1}n_{2}}\sum_{k} \frac{l_{kj}\rho_{gk}}{m_{k}}+\frac{n_{s}}{\surd n_{1}n_{2}}\rho_{gk}+ \frac{n_{s}}{\surd n_{1}n_{2}}\rho_{e}= \sqrt{n_{1}n_{2}}\sum_{k} \frac{l_{kj}\rho_{gk}}{m_{k}}+ \frac{n_{s}}{\surd n_{1}n_{2}}\rho.$ | (32) |
| --- | --- |

We finally have

| $\left( \begin{matrix} z_{1j} \\ z_{2j} \end{matrix} \right) \sim MVN\left( \begin{matrix} \left( \begin{matrix} 0 \\ 0 \end{matrix} \right) & \sum_{k=1}^{K} \frac{l_{kj}}{m_{k}}\left( \begin{matrix} h_{1k}^{2}n_{1} & \rho_{gk}\sqrt{n_{1}n_{2}} \\ \rho_{gk}\sqrt{n_{1}n_{2}} & h_{2k}^{2}n_{2} \end{matrix} \right)+\left( \begin{matrix} 1 & \frac{\rho n_{s}}{\sqrt{n_{1}n_{2}}} \\ \frac{\rho n_{s}}{\sqrt{n_{1}n_{2}}} & 1 \end{matrix} \right) \end{matrix} \right).$ | (33) |
| --- | --- |

**Extension Towards Controlling for Population Stratification**

Here, we extend GECKO to account for population stratification in the data. To do so, we need to make an additional assumption on how population stratification affects SNP-SNP correlation. To set up the intuition on what assumption to make, we follow the original paper of LDSC and first examine the effect of population stratification in the presence of two populations. Here, we denote $f_{1j}$ as the expectation of genotype for *j*’th SNP in population 1 in the first study, with

| $f_{1j}=E\left( x_{1ij} \vert i\in P_{1} \right).$ | (34) |
| --- | --- |

We assume that the first study consists of $n_{11}$ samples from population 1 and $n_{12}$ samples from population 2. Due to genotype centering, the expectation of genotype for *j*’th SNP in population 2 in the first study is

| $f_{2j}=E\left( x_{1ij} \vert i\in P_{2} \right)= -\frac{n_{11}}{n_{12}}f_{1j}.$ | (35) |
| --- | --- |

Similarly, we denote $f_{1j}^{'}$ as the expectation of genotype for *j*’th SNP in population 1 in the second study, with

| $f_{1j}^{'}=E\left( x_{2ij} \vert i\in P_{1} \right).$ | (36) |
| --- | --- |

We assume that the second study consists of $n_{21}$ samples from the population 1 and $n_{22}$ samples from population 2. Due to genotype centering, the expectation of genotype for *j*’th SNP in population 2 in the second study is

| $f_{2j}^{'}=E\left( x_{2ij} \vert i\in P_{2} \right)= -\frac{n_{21}}{n_{22}}f_{1j}^{'}.$ | (37) |
| --- | --- |

If there is no LD between the *j*’th and *k*’th SNPs, $cov\left( x_{1ij},x_{1ik} \right)=0$ in the absence of population stratification. However, population stratification induces additional SNP-SNP covariance as

| $cov\left( x_{1ij},x_{1ik} \right)=E\left( x_{1ij}x_{1ik} \right)-E\left( x_{1ij} \right)E\left( x_{1ik} \right)=\frac{n_{11}}{n_{1}}*f_{1j}f_{1k}+\frac{n_{12}}{n_{1}}\left( -\frac{n_{11}}{n_{12}}f_{1j}*\left( -\frac{n_{11}}{n_{12}}f_{1k} \right) \right)=\frac{n_{11}}{n_{12}}f_{1j}f_{1k},$ | (38) |
| --- | --- |

which is a scalar and can be defined as

$$\alpha_{1jk}= \frac{n_{11}}{n_{12}}f_{1j}f_{1k}.$$

While the above derivation is based on two populations, the fact that population stratification induces additional SNP-SNP covariance is rather general. Therefore, we make the following modeling assumption to extend GECKO in the presence of population stratification:

| $cov\left( x_{1ij},x_{1ik} \right) \sim MVN\left( \begin{matrix} \left( \begin{matrix} 0 \\ 0 \end{matrix} \right) & \left( \begin{matrix} 1 & \gamma_{jk}+\alpha_{1jk} \\ \gamma_{jk}+\alpha_{1jk} & 1 \end{matrix} \right) \end{matrix} \right),$  $cov\left( x_{2ij},x_{2ik} \right) \sim MVN\left( \begin{matrix} \left( \begin{matrix} 0 \\ 0 \end{matrix} \right) & \left( \begin{matrix} 1 & \gamma_{jk}+\alpha_{2jk} \\ \gamma_{jk}+\alpha_{2jk} & 1 \end{matrix} \right) \end{matrix} \right).$ | (39) |
| --- | --- |

where the first covariance term $\gamma_{jk}$ is due to LD between the two SNPs while second terms $\alpha_{1jk}$ and $\alpha_{2jk}$ are induced by population stratifications in the two studies, respectively. With the above assumptions, we have

| $E\left[ z_{1j} \right]=\frac{E(X_{1j}^{T}y_{1})}{\surd n_{1}}= \frac{E(X_{1j}^{T}\left( X_{1}\beta_{1}+\epsilon_{1} \right))}{\surd n_{1}}= 0,$  $E\left[ z_{2j} \right]=\frac{E(X_{2j}^{T}y_{2})}{\surd n_{2}}= \frac{E(X_{2j}^{T}\left( X_{2}\beta_{2}+\epsilon_{2} \right))}{\surd n_{2}}= 0.$ | (40) |
| --- | --- |

The covariance structure of $z_{1j}$, $z_{2j}$ is derived as following,

| $var\left( z_{1j} \right)=E\left( z_{1j}^{2} \right)-E\left( z_{1j} \right)^{2}$ $=\frac{E\left( X_{1j}^{T}\left( X_{1}\beta_{1}+\epsilon_{1} \right)\left( X_{1}\beta_{1}+\epsilon_{1} \right)^{T}X_{1j} \right)}{n_{1}}$ $=\frac{h_{1}^{2}}{n_{1}m}E\left[ X_{1j}^{T}X_{1}X_{1}^{T}X_{1j} \right]+\frac{\left( 1-h_{1}^{2} \right)}{n_{1}}E\left( X_{1j}^{T}X_{1j} \right).$ | (41) |
| --- | --- |

Where,

| $E\left[ X_{1j}^{T}X_{1}X_{1}^{T}X_{1j} \right]=E\left[ \sum_{k=1}^{m} X_{1j}^{T}X_{1k}X_{1k}^{T}X_{1j} \right]$ $=E\left[ \sum_{k=1}^{m} (\sum_{i=1}^{n_{1}} x_{1ij}x_{1ik})(\sum_{i=1}^{n_{1}} x_{1ij}x_{1ik}) \right]$ $=E\left[ \sum_{k=1}^{m} \left( \sum_{i=1}^{n_{1}} x_{1ij}^{2}x_{1ik}^{2}+\sum_{i\neq i^{'}} x_{1ij}x_{1ik}x_{1i^{'}j}x_{1i^{'}k} \right) \right]$ $=\sum_{k=1}^{m} E\left( \sum_{i=1}^{n_{1}} x_{1ij}^{2}x_{1ik}^{2}+\sum_{i\neq i^{'}} x_{1ij}x_{1ik}x_{1i^{'}j}x_{1i^{'}k} \right).$ | (42) |
| --- | --- |

The first term in equation (42) is

$$E{(x}_{1ij}^{2}x_{1ik}^{2})={E{(x}_{1ij}x_{1ik})}^{2}+var{(x}_{1ij}x_{1ik}),$$

$${E{(x}_{1ij}x_{1ik})}^{2}=\left( \gamma_{jk}+\alpha_{1jk} \right)^{2},$$

where

$$var{(x}_{1ij}x_{1ik})=E\left( var{(x}_{1ij}x_{1ik}\left| x_{1ik} \right) \right)+var\left( E\left( {(x}_{1ij}x_{1ik}\left| x_{1ik} \right) \right) \right).$$

Consequently,

$$E\left( var{(x}_{1ij}x_{1ik}\left| x_{1ik} \right) \right)=E\left( x_{1ik}^{2}\left( 1-\left( \gamma_{jk}+\alpha_{jk} \right)^{2} \right) \right)=1-\left( \gamma_{jk}+\alpha_{1jk} \right)^{2},$$

$$var\left( E\left( {(x}_{1ij}x_{1ik}\left| x_{1ik} \right) \right) \right)=var\left( x_{1ik}^{2}r_{jk} \right)=2\left( \gamma_{jk}+\alpha_{1jk} \right)^{2},$$

| $E{(x}_{1ij}^{2}x_{1ik}^{2})={E{(x}_{1ij}x_{1ik})}^{2}+var{(x}_{1ij}x_{1ik})= 1+2\left( \gamma_{jk}+\alpha_{1jk} \right)^{2}.$ | (43) |
| --- | --- |

By Isserlis’ thereom, the second term in equation (42) could be expanded as follows

$$E{(x}_{1ij}x_{1ik}x_{1i^{'}j}x_{1i^{'}k})$$

$$=E{(x}_{1ij}x_{1ik})E(x_{1i^{'}j}x_{1i^{'}k})+E{(x}_{1ij}x_{1i^{'}j})E(x_{1ik}x_{1i^{'}k})+E{(x}_{1ij}x_{1i^{'}k})E(x_{1ik}x_{1i^{'}j})$$

$$=\left( r_{jk}+\alpha_{1jk} \right)^{2},$$

$$E{(x}_{1ij}x_{1ik})={E\left( x_{1i^{'}j}x_{1i^{'}k} \right)=r}_{jk}+\alpha_{1jk},$$

$$E{(x}_{1ij}x_{1i^{'}j})= E\left( x_{1ik}x_{1i^{'}k} \right)=E{(x}_{1ij}x_{1i^{'}k})= E\left( x_{1ik}x_{1i^{'}j} \right)=0,$$

Therefore

| $E{(x}_{1ij}x_{1ik}x_{1i^{'}j}x_{1i^{'}k})= \left( r_{jk}+\alpha_{1jk} \right)^{2}.$ | (44) |
| --- | --- |

Combined equation (43) and (44), we have

| $E\left[ X_{1j}^{T}X_{1}X_{1}^{T}X_{1j} \right]\approx(\sum_{k=1}^{m} n_{1}^{2}\left( \gamma_{jk}+\alpha_{1jk} \right)^{2})+n_{1}m$ $=\left( n_{1}^{2}\sum_{k=1}^{m} r_{jk}^{2}+2r_{jk}\alpha_{1jk}+\alpha_{1jk}^{2} \right)+n_{1}m.$ | (45) |
| --- | --- |

$$E\left( X_{1j}^{T}X_{1j} \right)=E\left[ \sum_{i=1}^{n_{1}} x_{1ij}^{2} \right]=n_{1}$$

Therefore,

| $var\left( z_{1j} \right)= \frac{h_{1}^{2}}{n_{1}m}E\left[ X_{1j}^{T}X_{1}X_{1}^{T}X_{1j} \right]+\frac{\left( 1-h_{1}^{2} \right)}{n_{1}}E\left( X_{1j}^{T}X_{1j} \right)= \frac{h_{1}^{2}}{n_{1}m}\left[ \left( n_{1}^{2}\sum_{k=1}^{m} r_{jk}^{2}+2r_{jk}\alpha_{1jk}+\alpha_{1jk}^{2} \right)+n_{1}m \right]+ \frac{\left( 1-h_{1}^{2} \right)}{n_{1}}*n_{1}=\frac{n_{1}h_{1}^{2}}{m}l_{j}+1+(\frac{2n_{1}h_{1}^{2}}{m}\sum_{k=1}^{m} r_{jk}\alpha_{1jk}+\frac{n_{1}h_{1}^{2}}{m}\sum_{k=1}^{m} \alpha_{1jk}^{2}).$ | (46) |
| --- | --- |

Similarly, we could get,

| $var\left( z_{2j} \right)= \frac{n_{2}h_{2}^{2}}{m}l_{j}+1+\left( \frac{2n_{2}h_{2}^{2}}{m}\sum_{k=1}^{m} r_{jk}\alpha_{2jk}+\frac{n_{2}h_{2}^{2}}{m}\sum_{k=1}^{m} \alpha_{2jk}^{2} \right).$ | (47) |
| --- | --- |

For the covariance term, we denote $X_{1u}$ as the $\left( n_{1}-n_{s} \right)$ by *m* genotype matrix for the unique individuals in the first study and $X_{2u}$ as the $\left( n_{2}-n_{s} \right)$ by *m* genotype matrix for the unique individuals in the first study. Therefore, we have $X_{1}=\left( X_{s}, X_{1u} \right)$and $X_{2}=\left( X_{s}, X_{2u} \right)$. We further assume that,

| $ov\left( x_{sij},x_{sik} \right) \sim MVN\left( \begin{matrix} \left( \begin{matrix} 0 \\ 0 \end{matrix} \right) & \left( \begin{matrix} 1 & \gamma_{jk}+\alpha_{sjk} \\ \gamma_{jk}+\alpha_{1jk} & 1 \end{matrix} \right) \end{matrix} \right),$  $cov\left( x_{1uij},x_{1uik} \right) \sim MVN\left( \begin{matrix} \left( \begin{matrix} 0 \\ 0 \end{matrix} \right) & \left( \begin{matrix} 1 & \gamma_{jk}+\alpha_{1ujk} \\ \gamma_{jk}+\alpha_{1jk} & 1 \end{matrix} \right) \end{matrix} \right),$  $cov\left( x_{2uij},x_{2uik} \right) \sim MVN\left( \begin{matrix} \left( \begin{matrix} 0 \\ 0 \end{matrix} \right) & \left( \begin{matrix} 1 & \gamma_{jk}+\alpha_{2ujk} \\ \gamma_{jk}+\alpha_{1jk} & 1 \end{matrix} \right) \end{matrix} \right).$ | (48) |
| --- | --- |

Consequently, we have

| $cov\left( z_{1j}, z_{2j} \right)=E\left( z_{1j}z_{2j} \right)-E\left( z_{1j} \right)E\left( z_{2j} \right)=E(z_{1j}z_{2j})$  $E\left( z_{1j}z_{2j} \right)= \frac{1}{\sqrt{n_{1}n_{2}}}E\left( X_{1j}^{T}\left( X_{1}\beta_{1}+\epsilon_{1} \right)\left( X_{2}\beta_{2}+\epsilon_{2} \right)^{T}X_{2j} \right)$ $= \frac{1}{\sqrt{n_{1}n_{2}}}E\left( X_{1j}^{T}X_{1}\beta_{1}\beta_{2}^{T}X_{2}^{T}X_{2j}+X_{1j}^{T}\epsilon_{1}\epsilon_{2}^{T}X_{2j} \right)$ $=\frac{\rho_{g}}{m\sqrt{n_{1}n_{2}}}{E(X}_{1j}^{T}X_{1}X_{2}^{T}X_{2j})+ \frac{\rho_{e}}{\sqrt{n_{1}n_{2}}}{E(X}_{sj}^{T}X_{sj}).$ | (49) |
| --- | --- |

| $E\left[ X_{1j}^{T}X_{1}X_{2}^{T}X_{2j} \right]=E\left[ \left( X_{sj}, X_{1uj} \right)^{T}\left( X_{s}, X_{1u} \right) \left( X_{s},X_{2u} \right)^{T}\left( X_{sj},X_{2uj} \right) \right]$  $= E\left[ X_{sj}^{T}X_{s}X_{s}^{T}X_{sj}+X_{1uj}^{T}X_{1u}X_{2u}^{T}X_{2uj}+X_{1uj}^{T}X_{1u}X_{s}^{T}X_{sj}+X_{sj}^{T}X_{s}X_{2u}^{T}X_{2uj} \right]$ $=E\left[ \sum_{k=1}^{m} X_{sj}^{T}X_{sk}X_{sk}^{T}X_{sj}+X_{1uj}^{T}X_{1uk}X_{2uk}^{T}X_{2uj}+X_{1uj}^{T}X_{1uk}X_{sk}^{T}X_{sj}+X_{sj}^{T}X_{sk}X_{2uk}^{T}X_{2uj} \right].$ | (50) |
| --- | --- |

The first term in equation (50) is

| $E\left[ \sum_{k=1}^{m} X_{nsj}^{T}X_{nsk}X_{nsk}^{T}X_{nsj} \right] \approx(\sum_{k=1}^{m} n_{s}^{2}\left( \gamma_{jk}+\alpha_{sjk} \right)^{2})+n_{s}m$ $=\left( n_{s}^{2}\sum_{k=1}^{m} r_{jk}^{2}+2r_{jk}\alpha_{sjk}+\alpha_{sjk}^{2} \right)+n_{s}m.$ | (51) |
| --- | --- |

The second term in equation (50) is

$$E\left[ \sum_{k=1}^{m} X_{1uj}^{T}X_{1uk}X_{2uk}^{T}X_{2uj} \right]= E\left[ \sum_{k=1}^{m} \{(\sum_{i=1}^{n_{1s}} x_{1sij}x_{1sik})*(\sum_{i^{'}=1}^{n_{2s}} x_{2si^{'}j}x_{2si^{'}k})\} \right]=E\left[ \sum_{k=1}^{m} \sum_{i,i^{'}} x_{1sij}x_{1sik}x_{2si^{'}j}x_{2si^{'}k} \right],$$

By Isserlis’ thereom,

$$E\left[ x_{1sij}x_{1sik}x_{2si^{'}j}x_{2si^{'}k} \right]$$

$$=E\left[ x_{1sij}x_{1sik} \right]E\left[ x_{2si^{'}j}x_{2si^{'}k} \right]+E\left[ x_{1sij}x_{2si^{'}j} \right]E\left[ x_{1sik}x_{2si^{'}k} \right]+E\left[ x_{1sij}x_{2si^{'}k} \right]E\left[ x_{1sik}x_{2si^{'}j} \right]$$

$$=E\left[ x_{1sij}x_{1sik} \right]E\left[ x_{2sij}x_{2sik} \right],$$

$$E\left[ x_{1sij}x_{1sik} \right]E\left[ x_{2si^{'}j}x_{2si^{'}k} \right]={(\gamma}_{jk}+\alpha_{1ujk}){(\gamma}_{jk}+\alpha_{2ujk}),$$

$$E{(x}_{1sij}x_{2si^{'}j})=E\left[ x_{1sik}x_{2si^{'}k} \right]= E\left[ x_{1sij}x_{2si^{'}k} \right]= E\left[ x_{1sik}x_{2si^{'}j} \right]=0.$$

Therefore,

| $E\left[ \sum_{k=1}^{m} X_{1uj}^{T}X_{1uk}X_{2uk}^{T}X_{2uj} \right]= E\left[ \sum_{k=1}^{m} \{(\sum_{i=1}^{n_{1s}} x_{1sij}x_{1sik})*(\sum_{i^{'}=1}^{n_{2s}} x_{2si^{'}j}x_{2si^{'}k})\} \right]=\sum_{k=1}^{m} {(n}_{1}-n_{s}){{(n}_{2}-n}_{s}){(\gamma}_{jk}+\alpha_{1ujk}){(\gamma}_{jk}+\alpha_{2ujk})=\sum_{k=1}^{m} {(n}_{1}-n_{s})(n_{2}-n_{s})r_{jk}^{2}+ \sum_{k=1}^{m} {(n}_{1}-n_{s}){(n_{2}-n}_{s})\left( \alpha_{1ujk}+ \alpha_{2ujk} \right)\gamma_{jk}+ \sum_{k=1}^{m} {(n}_{1}-n_{s}){(n_{2}-n}_{s})\alpha_{1ujk}\alpha_{2ujk.}$ | (52) |
| --- | --- |

Similarly for the third and fourth term in equation (50) we have,

| $E\left[ \sum_{k=1}^{m} X_{1sj}^{T}X_{1sk}X_{nsk}^{T}X_{nsj} \right]=\sum_{k=1}^{m} {(n}_{1}-n_{s})n_{s}{(\gamma}_{jk}+\alpha_{1ujk}){(\gamma}_{jk}+\alpha_{sjk})= \sum_{k=1}^{m} \left( n_{1}-n_{s} \right)n_{s}r_{jk}^{2}+\sum_{k=1}^{m} \left( n_{1}-n_{s} \right)n_{s}\left( \alpha_{1ujk}+\alpha_{sjk} \right)r_{jk}+\sum_{k=1}^{m} \left( n_{1}-n_{s} \right)n_{s}\alpha_{1ujk}\alpha_{sjk}.$ | (53) |
| --- | --- |
| $E\left[ \sum_{k=1}^{m} X_{2sj}^{T}X_{2sk}X_{nsk}^{T}X_{nsj} \right]=\sum_{k=1}^{m} {(n}_{2}-n_{s})n_{s} {(\gamma}_{jk}+\alpha_{2ujk}){(\gamma}_{jk}+\alpha_{sjk})=\sum_{k=1}^{m} \left( n_{2}-n_{s} \right)n_{s}r_{jk}^{2}+\sum_{k=1}^{m} \left( n_{2}-n_{s} \right)n_{s}\left( \alpha_{2ujk}+\alpha_{sjk} \right)r_{jk}+\sum_{k=1}^{m} \left( n_{2}-n_{s} \right)n_{s}\alpha_{2ujk}\alpha_{sjk}.$ | (54) |

| $E\left[ X_{1j}^{T}X_{1}X_{2}^{T}X_{2j} \right]= n_{1}n_{2}\sum_{k=1}^{m} r_{jk}^{2}+n_{s}m+\sum_{k=1}^{m} r_{jk}(2n_{s}^{2}\alpha_{sjk}+{(n}_{1}-n_{s}){(n_{2}-n}_{s})(\alpha_{1ujk}+ \alpha_{2ujk})+\left( n_{1}-n_{s} \right)n_{s}\left( \alpha_{1ujk}+\alpha_{sjk} \right)+\left( n_{2}-n_{s} \right)n_{s}\left( \alpha_{2ujk}+\alpha_{sjk} \right))+\sum_{k=1}^{m} n_{s}^{2}\alpha_{sjk}^{2}+{(n}_{1}-n_{s}){(n_{2}-n}_{s})\alpha_{1ujk}\alpha_{2ujk}+\left( n_{1}-n_{s} \right)n_{s}\alpha_{1ujk}\alpha_{sjk}+\left( n_{2}-n_{s} \right)n_{s}\alpha_{2ujk}\alpha_{sjk}.$ | (55) |
| --- | --- |

Combined equations (51-54) together we could have,

| $E\left( z_{1j}z_{2j} \right)= \frac{1}{\sqrt{n_{1}n_{2}}}E\left( X_{1j}^{T}X_{1}\beta_{1}\beta_{2}^{T}X_{2}^{T}X_{2j}+X_{1j}^{T}\epsilon_{1}\epsilon_{2}^{T}X_{2j} \right)=\frac{\rho_{g}}{m\sqrt{n_{1}n_{2}}}{E(X}_{1j}^{T}X_{1}X_{2}^{T}X_{2j})+ \frac{\rho_{e}}{\sqrt{n_{1}n_{2}}}{E(X}_{sj}^{T}X_{sj})= \frac{\rho_{g}}{m\sqrt{n_{1}n_{2}}}*\left( n_{1}n_{2}\sum_{k=1}^{m} r_{jk}^{2}+n_{s}m+\sum_{k=1}^{m} r_{jk}(2n_{s}^{2}\alpha_{sjk}+{(n}_{1}-n_{s}){(n_{2}-n}_{s})(\alpha_{1ujk}+ \alpha_{2ujk})+\left( n_{1}-n_{s} \right)n_{s}\left( \alpha_{1ujk}+\alpha_{sjk} \right)+\left( n_{2}-n_{s} \right)n_{s}\left( \alpha_{2ujk}+\alpha_{sjk} \right))+\sum_{k=1}^{m} n_{s}^{2}\alpha_{sjk}^{2}+{(n}_{1}-n_{s}){(n_{2}-n}_{s})\alpha_{1ujk}\alpha_{2ujk}+\left( n_{1}-n_{s} \right)n_{s}\alpha_{1ujk}\alpha_{sjk}+\left( n_{2}-n_{s} \right)n_{s}\alpha_{2ujk}\alpha_{sjk} \right)+\frac{\rho_{e}n_{s}}{\sqrt{n_{1}n_{2}}}= \frac{\rho_{g}\sqrt{n_{1}n_{2}}}{m}l_{j}+\frac{n_{s}\rho}{\sqrt{n_{1}n_{2}}}+\frac{\rho_{g}}{m\sqrt{n_{1}n_{2}}}*\left( \sum_{k=1}^{m} r_{jk}(2n_{s}^{2}\alpha_{sjk}+{(n}_{1}-n_{s}){(n_{2}-n}_{s})(\alpha_{1ujk}+ \alpha_{2ujk})+\left( n_{1}-n_{s} \right)n_{s}\left( \alpha_{1ujk}+\alpha_{sjk} \right)+\left( n_{2}-n_{s} \right)n_{s}\left( \alpha_{2ujk}+\alpha_{sjk} \right))+\sum_{k=1}^{m} n_{s}^{2}\alpha_{sjk}^{2}+{(n}_{1}-n_{s}){(n_{2}-n}_{s})\alpha_{1ujk}\alpha_{2ujk}+\left( n_{1}-n_{s} \right)n_{s}\alpha_{1ujk}\alpha_{sjk}+\left( n_{2}-n_{s} \right)n_{s}\alpha_{2ujk}\alpha_{sjk} \right).$ | (56) |
| --- | --- |

Therefore,

We define

$Intercept_{1}= 1+(\frac{2n_{1}h_{1}^{2}}{m}\sum_{k=1}^{m} r_{jk}\alpha_{1jk}+\frac{n_{1}h_{1}^{2}}{m}\sum_{k=1}^{m} \alpha_{1jk}^{2})$,

$Intercept_{2}= 1+(\frac{2n_{2}h_{2}^{2}}{m}\sum_{k=1}^{m} r_{jk}\alpha_{2jk}+\frac{n_{2}h_{2}^{2}}{m}\sum_{k=1}^{m} \alpha_{2jk}^{2})$,

$$Intercept_{cov}= \frac{\rho_{g}}{m\sqrt{n_{1}n_{2}}}*\left( \sum_{k=1}^{m} r_{jk}(2n_{s}^{2}\alpha_{sjk}+{(n}_{1}-n_{s}){(n_{2}-n}_{s})(\alpha_{1ujk}+ \alpha_{2ujk})+\left( n_{1}-n_{s} \right)n_{s}\left( \alpha_{1ujk}+\alpha_{sjk} \right)+\left( n_{2}-n_{s} \right)n_{s}\left( \alpha_{2ujk}+\alpha_{sjk} \right))+\sum_{k=1}^{m} n_{s}^{2}\alpha_{sjk}^{2}+{(n}_{1}-n_{s}){(n_{2}-n}_{s})\alpha_{1ujk}\alpha_{2ujk}+\left( n_{1}-n_{s} \right)n_{s}\alpha_{1ujk}\alpha_{sjk}+\left( n_{2}-n_{s} \right)n_{s}\alpha_{2ujk}\alpha_{sjk} \right).$$

Therefore, the joint distribution for the two marginal z-scores of the *j*’th SNP is

| $\left( \begin{matrix} z_{1j} \\ z_{2j} \end{matrix} \right) \sim MVN\left( \begin{matrix} \left( \begin{matrix} 0 \\ 0 \end{matrix} \right) & \frac{l_{j}}{m}\left( \begin{matrix} h_{1}^{2}n_{1} & \rho_{g}\sqrt{n_{1}n_{2}} \\ \rho_{g}\sqrt{n_{1}n_{2}} & h_{2}^{2}n_{2} \end{matrix} \right) +\left( \begin{matrix} Intercept_{1} & \frac{\rho n_{s}}{\sqrt{n_{1}n_{2}}}+Intercept_{cov} \\ \frac{\rho n_{s}}{\sqrt{n_{1}n_{2}}}+Intercept_{cov} & Intercept_{2} \end{matrix} \right) \end{matrix} \right).$ | (57) |
| --- | --- |

The equation (57) relies on three intercept terms to model population stratification, thus effectively extending the way of modeling population stratification in the univariate LDSC towards multivariate modeling of two traits. Clearly, due to population stratification, the second covariance term becomes a summation of the environmental covariance and three intercept terms determined by several parameters ($\alpha_{1jk}$, $\alpha_{2jk}$, $\alpha_{sjk}$, $\alpha_{1ujk}$, $\alpha_{2ujk}$) that characterize the effect of population stratification on SNP-SNP covariance in the corresponding samples. The large number of parameters introduced due to population stratification unfortunately lead to model identifiability issues and render it impossible to estimate the environmental covariance. However, the genetic covariance can still be estimated in the presence of population stratification. In addition, if we further make the assumption that all introduced parameters equal to each other $\alpha_{1jk}=\alpha_{2jk}=\alpha_{sjk}=\alpha_{1ujk}=\alpha_{2ujk}=\alpha$, and $\sum\gamma_{jk}=0$, then we have

$Intercept_{1}= 1+n_{1}h_{1}^{2}\alpha^{2}$,

$Intercept_{2}= 1+n_{2}h_{2}^{2}\alpha^{2}$,

$$Intercept_{cov}= \rho_{g}\sqrt{n_{1}n_{2}}\alpha^{2}.$$

Therefore,

| $\left( \begin{matrix} z_{1j} \\ z_{2j} \end{matrix} \right) \sim MVN\left( \begin{matrix} \left( \begin{matrix} 0 \\ 0 \end{matrix} \right) & \frac{l_{j}}{m}\left( \begin{matrix} h_{1}^{2}n_{1} & \rho_{g}\sqrt{n_{1}n_{2}} \\ \rho_{g}\sqrt{n_{1}n_{2}} & h_{2}^{2}n_{2} \end{matrix} \right) +\left( \begin{matrix} 1+n_{1}h_{1}^{2}\alpha^{2} & \frac{\rho n_{s}}{\sqrt{n_{1}n_{2}}}+\rho_{g}\sqrt{n_{1}n_{2}}\alpha^{2} \\ \frac{\rho n_{s}}{\sqrt{n_{1}n_{2}}}+\rho_{g}\sqrt{n_{1}n_{2}}\alpha^{2} & 1+n_{2}h_{2}^{2}\alpha^{2} \end{matrix} \right) \end{matrix} \right)$. | (58) |
| --- | --- |

Consequently, the environmental covariance $\rho_{e}$ becomes identifiable from the parameter $\alpha$ for modeling population stratification. In this case, we just need to treat $\alpha$ as a separate parameter and proceed with the inference for $\rho_{g}$ and $\rho_{e}$ similar to what is detailed below.

**Optimization Algorithms**

**Notations**

To simplify notation, we reparametrize the model in equation (12) as

$$z_{j}=g_{j}^{*}+\epsilon_{j}^{*},$$

where

$$g_{j}^{*}\sim MVN\left( \begin{matrix} \left( \begin{matrix} 0 \\ 0 \end{matrix} \right) & \frac{l_{j}}{m}\left( \begin{matrix} h_{1}^{2}n_{1} & \rho_{g}\sqrt{n_{1}n_{2}} \\ \rho_{g}\sqrt{n_{1}n_{2}} & h_{2}^{2}n_{2} \end{matrix} \right) \end{matrix} \right),$$

$$\epsilon_{j}^{*}\sim MVN\left( \begin{matrix} \left( \begin{matrix} 0 \\ 0 \end{matrix} \right) & \left( \begin{matrix} 1 & \frac{\rho n_{s}}{\sqrt{n_{1}n_{2}}} \\ \frac{\rho N_{s}}{\sqrt{n_{1}n_{2}}} & 1 \end{matrix} \right) \end{matrix} \right).$$

We further define $V_{g} = \frac{l_{j}}{m}\left( \begin{matrix} h_{1}^{2}n_{1} & \rho_{g}\sqrt{n_{1}n_{2}} \\ \rho_{g}\sqrt{n_{1}n_{2}} & h_{2}^{2}n_{2} \end{matrix} \right)$ and $V_{e} =\left( \begin{matrix} 1 & \frac{\rho n_{s}}{\sqrt{n_{1}n_{2}}} \\ \frac{\rho n_{s}}{\sqrt{n_{1}n_{2}}} & 1 \end{matrix} \right)$. Here, $V_{g}$ contains the genetic covariance parameter $\rho_{g}$ and $V_{e}$ contains the phenotypic covariance parameter $\rho$ which can be used to obtain the environmental covariance parameter $\rho_{e}$.

Below we describe the detailed optimization algorithms to obtain the above parameters. Our optimization procedure consists of three steps: a golden section search method for finding good initial values [2]; an expectation maximization (EM) algorithm to achieve stable optimization [3]; and finally a Newton-Raphson (NR) algorithm to obtain the final estimates [4,5]. We describe the three steps in details below. The golden section search provides accurate initial values, while the combined EM and NR algorithm takes advantages of the stability of EM algorithm (every iteration increases the likelihood) and the faster convergence of NR algorithm.

**Golden Section Search for Initial Values**

Parameters in $V_{g}$ and $V_{e}$ are initialized through golden section search by maximizing the marginal composite likelihood. We set the search space for the heritability parameters ($h_{1}^{2}, h_{2}^{2}$) to be from 0 to 1, and set the search space for the correlation parameters $(\rho_{g},\rho_{e})$ to be from -1 to 1. We updated these parameters using the golden section algorithm.

**EM Algorithm**

Recall that our composite likelihood is in the following form

$$\prod_{j=1}^{p} P\left( z_{j},g_{j}^{*} | V_{g},V_{e} \right)^{\frac{1}{l_{j}}}=\prod_{j=1}^{p} (P\left( z_{j} | g_{j}^{*},V_{g},V_{e} \right)*P{\left( g_{j}^{*} | V_{g} \right))}^{\frac{1}{l_{j}}}.$$

Therefore, after ignoring the constants, the log composite likelihood is

| $\sum_{j=1}^{p} \mathrm{Log}P\left( z_{j},g_{j}^{*} \vert V_{g},V_{e} \right)=-\sum_{j=1}^{p} \frac{1}{{2l}_{j}}(log\left\vert V_{e} \right\vert+\left( \left( z_{j}-g_{j}^{*} \right)^{T}V_{e}^{-1}\left( z_{j}-g_{j}^{*} \right) \right)+log\left\vert V_{g} \right\vert+\left( g_{j}^{*}V_{g}^{-1}g_{j}^{*} \right)).$ | (59) |
| --- | --- |

We treat $g_{j}^{*}$ as missing values and derive the EM algorithm for parameter estimation. The EM algorithm consists of two steps: the expectation step and the maximization step.

In the expectation step, we consider the expected log composite likelihood

$$Q = -E(\sum_{j=1}^{p} \frac{1}{{2l}_{j}}(log\left| V_{e} \right|+\left( \left( z_{j}-g_{j}^{*} \right)^{T}Ve^{-1}\left( z_{j}-g_{j}^{*} \right) \right)+log\left| V_{g} \right|+\left( g_{j}^{*}V_{g}^{-1}g_{j}^{*} \right))),$$

where the expectation is taken with respect to the conditional distribution ${g_{j}}^{*},{\epsilon_{j}}^{*}|V_{g},V_{e},z_{j}$

| $g_{j}^{*}\vert z_{j}, V_{g}, V_{e} \sim MVN\left( V_{g}\Sigma^{-1}z_{j}, V_{g}-V_{g}\Sigma^{-1}V_{g} \right),$ | (60) |
| --- | --- |

| $\epsilon_{j}^{*}\vert z_{j}, V_{g}, V_{e} \sim MVN\left( V_{e}\Sigma^{-1}z_{j}, V_{e}-V_{e}\Sigma^{-1}V_{e} \right).$ | (61) |
| --- | --- |

In the maximization step, we maximize the Q function by setting its first order derivatives to be zero. We obtain

$$\hat{V_{g}}=\frac{\sum_{j=1}^{p} \frac{1}{l_{j}}E_{g_{j}^{*}|z_{j}}\left( g_{j}^{*}{g_{j}^{*}}^{T} \right)}{\sum_{j=1}^{p} \frac{1}{l_{j}}},$$

$$\hat{V_{e}}=\frac{\sum_{j=1}^{p} \frac{1}{l_{j}}E_{\epsilon_{j}|z_{j}}\left( {\epsilon_{j}}^{*}{{\epsilon_{j}}^{*}}^{T} \right)}{\sum_{j=1}^{p} \frac{1}{l_{j}}}.$$

We use the conditional distribution of ${g_{j}}^{*}|z_{j}$in equation (60) to estimate $E\left( g_{j}^{*}{g_{j}^{*}}^{T} \right)$ and the conditional distribution of ${\epsilon_{j}}^{*}|z_{j}$in equation (61) to $\mathrm{estimate}E\left( {\epsilon_{j}}^{*}{{\epsilon_{j}}^{*}}^{T} \right)$. Therefore,

$$E\left( g_{j}g_{j}^{T} | z \right)=V_{g}\Sigma_{j}^{-1}z_{j}z_{j}^{T}\Sigma_{j}^{-1}V_{g}+V_{g}-V_{g}\Sigma_{j}^{-1}V_{g},$$

$$E\left( e_{j}e_{j}^{T}|z \right)=V_{e}\Sigma_{j}^{-1}z_{j}z_{j}^{T}\Sigma_{j}^{-1}V_{e}+V_{e}-V_{e}\Sigma_{j}^{-1}V_{e}.$$

We performed EM as described above with 200 iterations or until the log likelihood increase between two consecutive iterations is below ${10}^{-6}$.

**Newton-Raphson Algorithm**

We denote $\Sigma=V_{g}+V_{e}$ and we can re-write the log composite likelihood function in equation (59) as

$$l(V_{g},V_{e})=\sum_{j=1}^{p} \frac{1}{l_{j}}(-\frac{1}{2}log\left| V_{g} \right|-\frac{1}{2}\log\left| V_{e} \right|- \frac{1}{2}{{(z}_{j}-g_{j})}^{T}V_{e}^{-1}{(z}_{j}-g_{j})- \frac{1}{2}{g_{j}}^{T}V_{g}^{-1}g_{j}).$$

We also denote $\hat{g}_{j}=V_{g}\Sigma^{-1}z_{j}$ to further simplify the log composite likelihood function as

$$l\left( V_{g},V_{e} \right)=\sum_{j=1}^{p} \frac{1}{l_{j}}(-\frac{1}{2}log\left| V_{g} \right|-\frac{1}{2}\log\left| V_{e} \right|- \frac{1}{2}z_{j}^{T}\Sigma^{-1}z_{j}).$$

On the above equation, we take the first and second order derivatives to obtain the score function and information matrix, with which we use Newton-Raphson algorithm to perform optimization.

The first order derivatives are

$$\frac{\partial l\left( V_{g},V_{e} \right)}{\partial\left( V_{g} \right)_{i,j}}=\frac{1}{1+1_{i=j}}\{\sum_{j=1}^{p} -\frac{1}{2}trace\left( V_{g}^{-1}\left( I_{ij}+I_{ji} \right) \right)+ \frac{1}{2}z_{j}^{T}\Sigma^{-1}\left( I_{ij}+I_{ji} \right)\Sigma^{-1}z_{j}\},$$

$$\frac{\partial l\left( V_{g},V_{e} \right)}{\partial\left( V_{e} \right)_{i,j}}=\frac{1}{1+1_{i=j}}\{\sum_{j=1}^{p} -\frac{1}{2}trace\left( V_{e}^{-1}\left( I_{ij}+I_{ji} \right) \right)+ \frac{1}{2}z_{j}^{T}\Sigma^{-1}\left( I_{ij}+I_{ji} \right)\Sigma^{-1}z_{j}\}.$$

Therefore, the first order derivative of log-likelihood function is a six by one vector. Similarly, the second order derivatives are

$$\frac{\partial^{2}l\left( V_{g},V_{e} \right)}{\partial\left( V_{g} \right)_{i,j}\partial\left( V_{g} \right)_{i',j'}}=\frac{1}{1+1_{i=j}}\frac{1}{1+1_{i^{'}=j^{'}}}\left\{ \sum_{j=1}^{p} \frac{1}{2}trace\left( V_{g}^{-1}\left( I_{ij}+I_{ji} \right)V_{g}^{-1}\left( I_{i^{'}j^{'}}+I_{j^{'}i^{'}} \right) \right)+z_{j}^{T}\Sigma^{-1}\left( I_{ij}+I_{ji} \right)\Sigma^{-1}\left( I_{i^{'}j^{'}}+I_{j^{'}i^{'}} \right)\Sigma^{-1}z_{j} \right\},$$

$$\frac{\partial^{2}l\left( V_{g},V_{e} \right)}{\partial\left( V_{g} \right)_{i,j}\partial\left( V_{e} \right)_{i',j'}}=\frac{1}{1+1_{i=j}}\frac{1}{1+1_{i^{'}=j^{'}}}\sum_{j=1}^{p} -z_{j}^{T}\Sigma^{-1}\left( I_{ij}+I_{ji} \right)\Sigma^{-1}\left( I_{i^{'}j^{'}}+I_{j^{'}i^{'}} \right)\Sigma^{-1}z_{j},$$

$$\frac{\partial^{2}l\left( V_{g},V_{e} \right)}{\partial\left( V_{e} \right)_{i,j}\partial\left( V_{e} \right)_{i',j'}}=\frac{1}{1+1_{i=j}}\frac{1}{1+1_{i^{'}=j^{'}}}\left\{ \sum_{j=1}^{p} \frac{1}{2}trace\left( V_{e}^{-1}\left( I_{ij}+I_{ji} \right)V_{e}^{-1}\left( I_{i^{'}j^{'}}+I_{j^{'}i^{'}} \right) \right)+z_{j}^{T}\Sigma^{-1}\left( I_{ij}+I_{ji} \right)\Sigma^{-1}\left( I_{i^{'}j^{'}}+I_{j^{'}i^{'}} \right)\Sigma^{-1}z_{j} \right\},$$

which is a six by six matrix. NR used the EM output as initial and was updated by the first order derivative times the inverse of the second order derivative. We performed NR as described above with 100 iterations or until the absolute difference of log likelihood between two consecutive iterations is below ${10}^{-6}$.

**Standard Error**

We followed [6] and used block-wise Jackknife to compute the standard error for the genetic and environmental covariance estimates. To do so, we divided the genome into two hundred blocks with each block containing an equal number of SNPs. We removed one block at a time to perform Jackknife estimation and obtained standard errors. Afterwards, we constructed a Wald test to test the genetic and environmental covariances.

**References**

1. Zhou X. A unified framework for variance component estimation with summary statistics in genome-wide association studies. Ann Appl Stat. 2017;11(4):2027–51.

2. Chandrupatla TR. A new hybrid quadratic/bisection algorithm for finding the zero of a nonlinear function without using derivatives. Adv Eng Softw. 1997;28(3):145–9.

3. Kaimmalka R. Alteration de la fonction de reproduction chez Idotea balthica basteri (Crustacea, Isopoda) soumis a l’effet chronique d’un détergent non ionique [Internet]. Vol. 8, Marine Environmental Research. 1983. 41–62 p. Available from: https://www.jstor.org/stable/2984875%0Ahttp://linkinghub.elsevier.com/retrieve/pii/0141113683900466

4. Thompson R. The Estimation of Variance and Covariance Components with an Application when Records are Subject to Culling. Biometrics. 1973;29(3):527.

5. Patterson HD, Thompson R. Recovery of inter-block information when block sizes are unequal. Biometrika. 1971;58(3):545–54.

6. Efron B, Stein C. The Jackknife Estimate of Variance. Ann Stat. 1981;9(3):586–96.
