# Supplementary material for: Accurate genetic and environmental covariance estimation with composite likelihood in genome-wide association studies
Source: PLoS Genet. 2021 Jan 4;17(1):e1009293. doi: 10.1371/journal.pgen.1009293 (PMC7808654; doi:10.1371/journal.pgen.1009293)
Supplement: Attachment — Submitted filename: Point_to_Point_Response.docx [file pgen.1009293.s017.docx]

**Reviewer #1: This is a great paper with contributions to an important problem. Overall, the manuscript is well-structured and clearly written. Although I have some concerns and questions about the technical details of GECKO and some analyses presented in the paper, all my comments should be addressable in principle.**

**Response**: Thank you for your positive review and constructive comments. Our detailed responses are listed below.

**Major comments:**

**1. It was not immediately obvious to me if GECKO assumes the design matrix (X) to be fixed or random. Methods for genetic correlation estimation (and frankly, heritability estimation as well) have not been consistent on this issue. My recollection is that the GREML approach and GNOVA assume a fixed design while LDSC is based on a random design. While the field seems to have accepted that genetic correlation estimation is robust to these two different designs (at least empirically), whether this will affect the estimation of environmental covariance is unclear. Under a random design, the intercept of LDSC is the phenotypic covariance of two traits. Under a fixed design, I am under the impression that the intercept may actually be the environmental covariance instead. When the goal is to estimate the genetic covariance, such differences does not matter since the slope in LDSC is always a good estimator for rho_g. But the interpretation of intercept is completely different under two model assumptions. It will be important to clarify the assumptions in GECKO and provide some discussions on whether the environmental covariance is robust to different assumptions**.

**Responses:** Thank you for the comments. Our previous derivations were indeed based on a random matrix design where the genotype matrix X is assumed to be random and its first and second moments are treated as population level parameters. Following your comments, we added additional derivations for the fixed matrix design where the genotype matrix X is assumed to be known. In the fixed matrix design, we further considered the setting where we had to rely on a reference panel to estimate the SNP correlations instead of directly computing them based on the data at hand. Through these derivations, we found that the per-SNP marginal likelihood remains the same regardless of whether the design is fixed or random. Therefore, the intercept in our method for both the fixed and random matrix designs is the phenotypic covariance of two traits, consistent with the random design derivation in the LDSC paper. The detailed derivation for the random matrix design is updated in S1 Text (lines 28-83 on pages 2-6), which is followed by detailed derivations for the fixed matrix design (lines 84-102 on pages 6-9). We have now clearly stated the detailed modeling assumptions made for both derivations. We also briefly mention these two matrix design assumptions in the Discussion (lines 622-625 on page 30).

Out of curiosity, we went back to the GNOVA paper and carefully examined their derivations. We found that the intercept in GNOVA, which is based on a fixed matrix design, indeed consists of only the environmental covariance of two traits. However, we also found that such result is based on using a biased form of variance estimate instead of an unbiased form as used in our derivations for GECKO. Specifically, the fourth equation on page 940 in GNOVA paper reads

$$\frac{1}{m_{j}}\left( X_{j}^{T}y_{1} \right)^{T}Z_{j}^{T}y_{2}= \sum_{i=1}^{K} \frac{\rho_{i}}{m_{i}m_{j}}tr\left( Z_{j}^{T}Z_{i}X_{i}^{T}X_{j} \right)+\frac{\rho_{e}}{m_{j}}\sum_{t=1}^{N_{s}} \left( X_{j}X_{j}^{T} \right)_{tt}, j=1,\ldots,k.$$

Above, *k* is the number of annotation categories. If we ignore the complication of functional annotations, the above equation is effectively

$$\frac{1}{\sqrt{n_{1}n_{2}}}z_{1j}z_{2j}=\frac{1}{\sqrt{n_{1}n_{2}}}\left( X_{j}^{T}y_{1} \right)^{T}Z_{j}^{T}y_{2}=\frac{1}{\sqrt{n_{1}n_{2}}} \frac{\rho_{g}}{m}tr\left( Z_{j}^{T}ZXX_{j} \right)+\frac{1}{\sqrt{n_{1}n_{2}}}\rho_{e}\sum_{t=1}^{N_{s}} \left( X_{j}X_{j}^{T} \right)_{tt}= \frac{\rho_{g}}{m\sqrt{n_{1}n_{2}}}\sum_{l=1} \tilde{r}_{jl}^{2}+\frac{\rho_{e}n_{s}}{\sqrt{n_{1}n_{2}}}.$$

where $\tilde{r}_{jl}^{2}$ is the sample R^2^ between the *j*’th and *l*’th SNPs. In the GNOVA paper, the sample $\tilde{r}_{jl}^{2}$ is replaced directly by the population level parameter $r_{jl}^{2}$. Consequently, the intercept term in GNOVA is $\frac{\rho_{e}n_{s}}{\sqrt{n_{1}n_{2}}}$, which is the environmental covariance instead of the phenotypic covariance. However, replacing the sample $\tilde{r}_{jl}^{2}$ with the population level parameter $r_{jl}^{2}$ is not ideal, as the sample covariance is not an unbiased estimate of the population covariance: $E\left[ \tilde{r}_{jl}^{2} \right]= r_{jl}^{2}+\frac{1}{n_{s}}$, with the bias being $\frac{1}{n_{s}}$. If we use the unbiased estimation of population covariance instead, then we need to sum $\frac{1}{n_{s}}$ across *m* different SNPs, leading to the addition of $\frac{\rho_{g}n_{s}}{\sqrt{n_{1}n_{2}}}$ to the intercept term to form the new intercept of$\frac{(\rho_{e}+\rho_{g})n_{s}}{\sqrt{n_{1}n_{2}}}$. Therefore, if we account for this bias term $\frac{1}{n_{s}}$, then the intercept from GNOVA would also have become the phenotypic covariance of the two traits.

**2. The field has not paid as much attention to the environmental covariance compared to genetic covariance. One reason is that the environmental covariance is more susceptible to technical artifacts (such as insufficiently controlled confounding) in the GWAS summary statistics. In fact, this was a highlight in genetic covariance estimation since if there are any technical correlations between two sets of GWAS, such correlations will most likely be independent from LD. The first LDSC paper on contrasting confounding from polygenicity is a good example on this issue (in the single-trait context). Suppose there is unadjusted population stratification in a GWAS, such inflation is randomly applied to SNPs in the genome while true polygenic effects are stronger in genomic regions with stronger LD. Therefore, all the technical bias will be absorbed into the intercept term of single-trait LDSC. The multi-trait extension of this problem is that technical correlation of two traits caused by population stratification will show up in the intercept of LDSC. I have been using LDSC to illustrate the problem since it's a famous method but I don't think GECKO is immune to the exact same issue. If you run two GWASs without adjusting for PCs, and apply GECKO to their sumstats, I expect the "environmental covariance" estimates to be severely biased while the genetic covariance estimates will remain solid. If the authors can come up with an approach to address this problem, it will improve this manuscript. Otherwise, at least treat this as a serious problem and list it as a limitation.**

**Responses:** Thank you for the comments. We completely agree with the reviewer that the estimation of environmental covariance is more susceptible to technical artifacts than the estimation of genetic covariance. Following your comments, we carried out additional model extensions and simulation analysis for the case of population stratification. Specifically, we followed the main idea of how the univariate LDSC controls for population stratification in heritability estimation and used an alternative strategy to extend such idea to GECKO to control for population stratification in covariance estimation. The extension of GECKO introduces three additional intercept parameters to control for population stratification that are present in the two unique samples of the two studies as well as in the common samples shared between the two studies, respectively. Such extension of GECKO effectively includes the univariate LDSC approach for controlling for population stratification using one intercept as a special case. Unfortunately, the environmental covariance cannot be directly estimated in the extended GECKO due to model identifiability issues, unless we make an additional modeling assumption that the same level of population stratification occurs in the unique and common samples. In this case, only one intercept is needed to control for population stratification, allowing us to solve the model identifiability issue and estimate the environmental covariance. The detailed model extension is provided in S1 Text (lines 170-279 on pages 14-26).

In addition, we followed the simulation strategy presented in the univariate LDSC paper to perform additional simulations to examine the performance of different methods under population stratification. Unfortunately, we were unable to reproduce the effective control of LDSC on population stratification in heritability estimation (details below) and instead observed similar upward biased heritability estimates for all three methods in the presence of population stratification. In addition, we observed upward biased genetic covariance estimates and downward biased environmental covariance estimates for all three methods in the simulations. We suspect these somewhat unexpected observations are due to the particular GWAS data we had for simulations, which is different from the GWAS data used in the LDSC paper. Therefore, we decided not to include these simulation results in the manuscript due to the preliminary nature of these results. Instead, we list them below for your reference. We would be more than happy to modify the simulations and include them into the manuscript if you identify any errors we might have made in the simulations.

We first describe the simulation details. Specifically, we used the WTCCC dataset for simulation as described in the Materials and Methods section. We used PLINK to obtain the top principle components from the genotype matrix. In each simulation, we first simulated the genetic effect sizes for all SNPs based on equation (2). We set the heritability of both traits to be 0.5, and we varied the genetic covariance value from -0.4 to 0.4 by a step of 0.2, with a fixed environmental covariance set to be 0, for genetic covariance estimation. We simulated the environmental effects based on equation (3). In addition, we varied environmental covariance value from -0.4 to 0.4 by a step of 0.2 with a fixed genetic covariance set to be 0, for environmental covariance estimation. We then added pc effects to the two traits. The sample variance of pc is $\frac{1}{n}=\frac{1}{2938}$. The small sample variance of the pc is due to the orthonormal constraint on each pc from PCA to ensure $\sum pc_{i}^{2}=1$. We varied the pc effects to be in the range of $\sqrt{0.5}\times\sqrt{2938}\times\sqrt{0.01}$ to $\sqrt{0.5}\times\sqrt{2938}\times\sqrt{0.1}$, so that pc effects account for 1%-10% of the total heritability of each trait. We then summed the genetic effects, the environmental effects and pc effects to obtain the simulated phenotypes based on equation (1). Afterwards, we computed marginal z-scores for each trait SNP pair using linear regression. We also computed LD scores using all individuals, with a window size set to be 1MB to minimize the influence of LD decay following the recommendation of LDSC. Finally, we applied different methods to estimate the genetic and environmental covariance based on the resulting z-scores and LD scores.

$$y_{1}=X_{1}\beta_{1}+pc*\alpha_{1}+\epsilon_{1},$$

$$y_{2}=X_{2}\beta_{2}+pc*\alpha_{2}+\epsilon_{2},$$

(1)

$$\left( \begin{matrix} \beta_{1j} \\ \beta_{2j} \end{matrix} \right)\sim MVN\left( \begin{matrix} \left( \begin{matrix} 0 \\ 0 \end{matrix} \right), & \frac{1}{m}\left( \begin{matrix} h_{1}^{2} & \rho_{g} \\ \rho_{g} & h_{2}^{2} \end{matrix} \right) \end{matrix} \right),$$

(2)

$$\left( \begin{matrix} \epsilon_{1i} \\ \epsilon_{2i} \end{matrix} \right)\sim MVN\left( \begin{matrix} \left( \begin{matrix} 0 \\ 0 \end{matrix} \right), & \left( \begin{matrix} 1-h_{1}^{2} & \rho_{e} \\ \rho_{e} & 1-h_{2}^{2} \end{matrix} \right) \end{matrix} \right),$$

(3)

We considered the population stratification setting under the one sample study design in simulations. We examined a total of 100 scenarios (1 study designs x 1 genetic covariance x 5 environmental covariance x 10 choices of pcs variability + 1 study designs x 1 environmental covariance x 5 genetic covariance x 10 choices of pcs variability).

We now describe our main observations. We first examined the estimation accuracy of heritability by the three methods for a range of pc effects (Figs 1A, B below). Note that the LDSC version we used contains an intercept for controlling for population stratification and the GECKO version we used is the extension of GECKO described above that also contains an intercept for controlling for population stratification. In the results, we found that all three methods produce a heritability estimate that is upward biased. The mean heritability estimates across various proportion of pc effects ranges from 0.61 to 1.18 for GECKO, 0.61 to 1.55 for LDSC, 0.58 to 1.34 for GNOVA. The bias of the heritability goes larger as the proportion of variance explained by pc increases (Fig 1A below). The estimation accuracy of GECKO is followed by LDSC with an MSE ratio ranging from 0.96 to 2.41. GNOVA is more accurate than GECKO when proportion of variance explained by pcs is small (1% - 7%), while GECKO is more accurate when proportion of variance explained by pcs is large (Fig 1B below).

We then examined the estimation accuracy of genetic covariance. All three methods also produce an estimate of the genetic covariance that is upward biased across range of true genetic covariance (-0.4 to 0.4). The bias of genetic covariance estimate increases as the proportion of variance explained by pc increases (Figs 1C, 1E, 1G, 1I, 1K below). We quantified the estimation accuracy in terms of MSE ratio given that all three methods provide biased estimate. GECKO has a more accurate estimate compared to GNOVA in all scenarios. LDSC has a more accurate estimate when true genetic covariance is -0.2 and 0 (Figs 1F and 1H below). GECKO outperforms LDSC when true genetic covariance is -0.4, 0.2 and 0.4 (Figs 1D, 1J and 1L below). The estimation accuracy is consistent with the observation without population stratifications. LDSC is more accurate when the summation of genetic and environmental covariance is zero, while GECKO is more accurate otherwise.

We next examined the environmental covariance estimate under population stratifications (Figs 2C-2L below). This time, all methods produce estimates of the environmental covariance that are downward biased (Figs 2C, 2E, 2I, 2G, 2K below). The bias increases as the proportion of variance explained by pc becomes larger. We examined the estimation accuracy of three methods in terms of MSE ratio. GECKO outperform GNOVA in all scenarios. LDSC performs better when true environmental covariance is -0.2, 0 (Figs 2F and 2H below), GECKO has a more accurate environmental covariance estimate when the true environmental covariance is -0.4, 0.2 and 0.4 (Figs 2D, 2J and 2L below).

As explained above, we were puzzled by these simulation results and thus did not include them in the updated manuscript. And we have followed the reviewer’s comment and add a short discussion on extension to the population stratification to the updated manuscript (lines 666-688 on page 32).

**Fig 1. Comparison of heritability and genetic covariance estimation of different methods under population stratification in simulations**. Compared methods include GECKO (purple), GNOVA (grey), and LDSC (green). Results are shown for heritability (A, B), genetic covariance (C - L). Boxplots display estimated heritability(A) and genetic covariances (C, E, G, I, K) on y-axis versus the 1%-10% proportion of the heritability explained by pcs on x-axis across simulation replicates. Estimation accuracy is measured by the ratio of mean square errors (MSE), which contrast the MSE from GNOVA or LDSC with respect to GECKO, across 1%-10% proportion of the heritability on x-axis, for heritability (B) and genetic covariances (D, F, H, J, L). An MSE ratio below one suggests that GECKO performs worse than the other method; above one otherwise.

**Fig 2. Comparison of heritability and environmental covariance estimation of different methods under population stratification in simulations**. Compared methods include GECKO (purple), GNOVA (grey), and LDSC (green). Results are shown for heritability (A, B), environmental covariance (C - L). Boxplots display estimated heritability(A) and environmental covariances (C, E, G, I, K) on y-axis versus 1%-10% proportion of the heritability explained by pcs on x-axis across simulation replicates. Estimation accuracy is measured by the ratio of mean square errors (MSE), which contrast the MSE from GNOVA or LDSC with respect to GECKO, across 1%-10% proportion of the heritability on x-axis, for heritability (B) and environmental covariances (D, F, H, J, L). An MSE ratio below one suggests that GECKO performs worse than the other method; above one otherwise.

**3. Only ~600k SNPs were used in the real data analyses which seems low. I wonder whether the somewhat heuristic composite likelihood approach in GECKO is still effective when there are dense SNPs with extensive LD in the analysis. This is important considering that both simulation and real data analysis in this paper were based on relatively low numbers of SNPs. LDSC also requires trimming of sumstats by taking the overlap with HAPMAP SNPs while GNOVA uses all available SNPs. After all, if there are few SNPs with minimal LD in the data, genetic and environmental covariance estimation becomes a much simpler problem. The tricky issue really is LD here.**

**Responses**: Thanks for the comments. Following your suggestions, we have performed additional simulations and real data analysis with a larger number of SNPs. Specifically, in the new simulations, we first imputed data based on the 1,000 Genomes project reference panel and used the imputed 1,819,851 SNPs for simulations (details in Method, lines 261-263 on page 13). The simulation results are largely consistent with the early results performed with ~600K SNPs. The detailed simulation results are provided in S6 Fig and described in Results (lines 485-488 on page 23).

Besides simulations, we also performed additional real data analysis. Analysis details are provided in the Method (lines 337-343 on page 16). Briefly, we focused on 13 (out of 22) phenotypes from GLGC and UKBB that have a higher number of SNPs than the remaining phenotypes. We used a common set of 1,731,189 SNPs available for these traits for analysis. Compared to the early analysis performed on 611,444 SNPs, the real data analysis with denser genotypes remains largely similar. For example, the spearman rank correlation on the genetic and environmental covariance estimates obtained from the two set of analyses is 0.865 and 0.98, respectively. Analysis on dense SNPs yielded slightly more significant genetic covariance pairs (37, vs 29 previously), with 28 (96.6%) pairs shared in common. In addition, a larger number of significant environmental covariance pairs were detected by analysis on dense SNPs (23, vs 15 previously), with 15 (100%) pairs shared in common. The detailed results are shown in Table S4 and are describes in Results (lines 605-619 on pages 28-29). Based on both simulations and the real data application, it appears that genotype density does not influence the qualitative conclusion and the ranking of the compared methods.

**4. It is helpful that GECKO can partition genetic covariance by annotation. It will be helpful if the authors can provide some discussions on whether the procedure can be easily generalized to overlapping annotations.**

**Response:** Thanks for the comment. Indeed, the procedure is easily generalized to overlapping annotations. Follow your comment, we have added detailed derivations for overlapping annotations in S1 Text (lines 145-168 on pages 11-14).

**5. The inflated type-I error of GNOVA illustrated in Table 1 is striking and also inconsistent with the original publication of GNOVA. However, these results are also inconsistent with what is shown in Figure 2. There, when the true genetic covariance is 0, all three approaches seem to have comparable type-I error rates. Why the contradiction?**

**Response**: Thanks for the comment. The results in Table 1 are actually not inconsistent with that shown in the GNOVA paper. Specifically, the GNOVA paper only shows results on the two-study design with no sample overlaps. GNOVA works well in the absence of sample overlaps as shown in both the GNOVA paper and Table 1 in our study. In contrast, the inflated type I error of GNOVA observed in Table 1 is for the one study design and the two-study design with sample overlaps, which was not examined in the GNOVA paper. We have added new explanations in the Results (lines 433-437 on pages 20-21) to clarify this issue.

In addition, the results in Table 1 are also not inconsistent with that in Figure 2. Specifically, the type I error results in Table 1 are based on a nominal p-value cutoff of 0.05. In contrast, the power results in Figure 2 are directly controlled at type I error rate of 0.05 but not nominal p-value of 0.05, to ensure a fair comparison among methods since some methods such as GNOVA does not have correct type I error control in certain settings. Therefore, the power should be exactly 0.05 in Figure 2 for all methods. We have added explanation in the Results (lines 440-441 on page 21) and Method (lines 279-285 on page 14) on this issue, and mentioned in the corresponding figure legend that Figure 2 is not based on a nominal p-value cutoff of 0.05 (lines 937-938 on page 39).

**Minor comments:**

**6. Figure 2's panels are labeled differently between the main text and the actual plot. See line 403 on page 24. I think the authors meant Fig2D and Fig2E instead of panels B and D.**

**Response**: Thanks for the comment. Indeed, it should be Fig2D and Fig2E instead of panels B and D. We have corrected this error in the updated manuscript (line 439 on page 21).

**7. In the real data analysis, is there any trait pair showing significant genetic and environmental covariance but with opposite signs? How to interest these results?**

**Response:** Thanks for the comment. We checked the results and did not find any trait pairs that show significant genetic and environmental covariance but with opposite signs. We did find that 9 trait pairs (out of 54 pairs) showed opposite signs of genetic and environmental covariance, but none of these pairs were significant. We briefly mentioned this point in the Results (lines 577-579 on page 27).

**8. Fig1E seems to suggest that LDSC had noisy estimates on genetic covariance but it contradicts Fig1F.**

**Response:** Thanks for the comment. We apologize for the incorrect coloring there. We previously incorrectly colored the GNOVA to green and LDSC to grey in Fig 1F. We have fixed this error in Fig1F in the updated manuscript.

**9. In the simulation corresponding to Fig2C, if we apply GECKO, should we expect to see near-null results with a "power" of 5%? In practice, it may not be obvious if two GWASs have overlapping samples. Consequently, it may not be easy (or even possible) to distinguish a lack of environmental covariance and a lack of sample overlap. It may be helpful to add some discussions on this issue.**

**Response:** Thank you for the comment. Indeed, it is often not obvious if two GWASs have overlapping samples and a careful examination needs to be carried out to determine the overlapping sample size. Because the overlapping sample size is coupled with the environmental covariance as the reviewer also brought up, the two parameters are not identifiable from each other. Therefore, it would be impossible to estimate the environmental covariance correctly if the overlapping sample size is unknown. We have added new explanations on this important issue, along with new simulations for demonstration. Simulation details are provided in the Method (lines 269-274 on page 13). Results are shown in S9 Fig and S10 Fig, with details provided in Results (lines 498-509 on pages 23-24)

**10. There have been some recent developments on genetic covariance estimation. For example, HDL (**[**https://www.nature.com/articles/s41588-020-0653-y**](https://www.nature.com/articles/s41588-020-0653-y)**) uses a likelihood-based approach to improve the efficiency of estimation and SUPERGNOVA (**[**https://www.biorxiv.org/content/10.1101/2020.05.08.084475v1.abstract**](https://www.biorxiv.org/content/10.1101/2020.05.08.084475v1.abstract)**) de-correlates the summary statistics first and then apply standard regression analysis. In addition, trans-ethnic estimation is an important topic and a clear future direction (**[**https://www.biorxiv.org/content/10.1101/803452v3**](https://www.biorxiv.org/content/10.1101/803452v3)**). Since these are very recent papers, it wouldn't be fair to ask for comparisons between GECKO and these methods. But some discussions regarding these new developments will improve the manuscript.**

**Response:** Thanks for the comment. We have followed the reviewer’s comment and add a short discussion on these important recent references to the updated manuscript (lines 630-639 on page 30).

**Reviewer #2: Review: Accurate genetic and environmental covariance estimation with composite likelihood in genome-wide association studies**

**Summary:
Gao et al introduce a novel statistical approach, GECKO, to estimate genetic and environmental covariance between complex traits using only GWAS summary statistics with a computationally efficient composite likelihood estimator. The approach is motivated by the lack of likelihood-based approaches in summary-statistic-based genetic covariance estimation (e.g., LDSC, GNOVA both use MoM estimators). The authors performed extensive simulations to validate their approach and demonstrate its improved performance. They then followed with application to real data as an illustration. The idea is interesting, and I found the manuscript extremely well written and straightforward to follow. I detail my main comments below.**

**Responses:** Thank you for your positive review and constructive comments. Our detailed responses are provided below.

**Major Comments:**

**1. The authors performed a considerable number of simulations demonstrating the mostly improved performance of GECKO with respect to LDSC and GNOVA. However, all simulations were performed essentially under the correct generative model, with no inspection of performance under model misspecification. A few scenarios come to mind:**

**a) LD pattens match exactly between the focal population and LD score computation. To investigate this point further, can the authors showcase the performance of all three methods when 1000G LD information is used to compute LD scores?**

**Response:** Thanks for the comment. Follow your suggestions, we have included several model misspecification scenarios. Specifically, for your comment 1a, we examined the case where the LD patterns do not match between the focal population and LD score computation, by using the genotype data from the 1,000 Genomes project as a reference panel to compute the LD scores. We found that the results are largely consistent with previous results and are not sensitive to the choice of the reference panel. The results are shown in S8 Fig and the Result (lines 492-498 on page 23), with simulation details provided in the Method (lines 266-270 on page 13).

**b) How does GECKO (and LDSC, GNOVA) perform under misspecification of n_s?**

**Response:** For your comment 1b, the parameter $n_{s}$ appears in the term $n_{s}\left( \rho_{e}+\rho_{g} \right)$, which is effectively treated as a parameter that is separated from $\rho_{g}$ in the composite likelihood (equation 12 in S1 Text). Therefore, misspecification of $n_{s}$ would not affect the estimation of the genetic covariance $\rho_{g}$. However, $n_{s}$ is coupled with the environmental covariance $\rho_{e}$ in the composite likelihood and these two parameters are not identifiable from each other. Consequently, under misspecification of $n_{s}$, the environmental covariance would not be estimated correctly: the environmental covariance will be overestimated if $n_{s}$ is underestimated and will be underestimated if $n_{s}$ is overestimated. The coupling/unidentifiability of $n_{s}$ and $\rho_{e}$ is a feature of multivariate linear mixed model and applies to all methods including GECKO, LDSC and GNOVA. We have added a paragraph in the Results (lines 498-509 on pages 23-24) on this important issue, with additional simulations for illustration. The detailed simulation details are provided in Method (lines 269-274 on page 13) with results shown in S9 Fig and S10 Fig.

**c) How does stratification affect the performance of GECKO? A straightforward simulation would be to generate both phenotypes as a function of several leading PCs, in addition to the remaining genetic + environmental model.**

**Response:** Population stratification turns out to be a complicated issue. Please refer to our response to reviewer #1’s comment #2 for details.

**Minor Comments:**

**1. MoM estimators are unbiased, and it is not unexpected to see estimates largely agree across all three methods. However, as the authors note, GECKO’s development was partly motivated by improving statistical power. The use of MSE ratios is interesting to get a relative sense of performance, but still find it difficult to judge what my expectation should be here (e.g., Is 1.05 really all that different). I appreciated the authors decision to include power calculations in the non-stratified setting given its clear interpretation but noticed their absence in the functional stratified simulations.**

**Response:** Thanks for the comment. Follow your suggestions, we have included power calculations for the simulations with stratified functional annotations. The results are largely consistent with the main results. The new simulation details are provided in the Method (lines 276-279 on pages 13-14), with results shown in S5 Fig and updated in the Results (lines 475-481 on pages 22-23). In addition, for your comment “The use of MSE ratios is interesting to get a relative sense of performance, but still find it difficult to judge what my expectation should be here (e.g., Is 1.05 really all that different)”, we note that MSE is a direct measure of the variance of the estimate. Because the variance of the estimate is a function of the sample size due to central limit theorem, the MSE ratio consequently implies how effective the two methods are in using the samples. For example, an MSE ratio of 1.11 implies that GECKO effectively increases the sample size by $1.11-1=11\%$. We included this interpretation of MSE in the Results (lines 365-369 on pages 17-18).

**2. Replace “*” with “x” in estimates/p-values.**

**Response:** Thanks for the comment. We have fixed this error in the updated manuscript.

**Reviewer #3: Gao et al. presented GECKO, a statistical method that aims to estimate both genetic and environmental covariances between different traits using GWAS summary statistics. The key idea of GECKO is to approximate complicated joint likelihood with a relatively simple composite likelihood, which holds the potential to be computationally fast if the weight parameters in the composite likelihood are correctly specified. The authors evaluated the performance of GECKO by simulations as well as the analysis of 22 traits collected from five large scale GWASs.**

**Response**: Thank you for your review and constructive comments. Our detailed point by point responses are listed below.

Main comments:

**1. Simulations: While the authors have simulated >200 scenarios, these mostly reflect the small step size used to evaluate the impact of genetic covariance (-0.4 to 0.4) or environmental covariance (-0.4 to 0.4), whereas the other key parameter, e.g., heritability, which is also extremely important, was set at constant values. The values used in the simulations do not seem to reflect those seen in real data that the authors showed later on. Overall, I feel that the current parameter values used in simulations are too different from what one would expect from real data, which make it difficult to judge whether these simulation results are useful. To get a better sense on the performance of GECKO, I think it is important to simulate data that are more representative of real data. I would suggest the authors to do the following:**

a) **Estimate heritability for the 22 traits collected from real GWASs. Estimate genetic covariances and environmental covariances using these real data. Use these estimates as a guideline to determine the range of parameter values for simulations.**

**Response**: Thanks for the comment. Our previously simulation settings actually followed the standard simulation settings commonly used in the field and were almost identical to what was done in the LDSC and GNOVA papers. Specifically, the LDSC paper used a single simulation setting with heritability of both traits set to be 0.58 and the genetic covariance set to be 0.29 (which corresponds to a genetic correlation of 0.5). The GNOVA paper used five simulation settings, with the heritability of both traits set to be 0.5 and the genetic covariance set to range from 0 to 0.25 (which corresponds to a genetic correlation ranging from 0 to 0.5) [1,2]. Therefore, following these studies and further extending beyond the settings examined in these studies, we previously set the heritability of both traits to be 0.5 and set the genetic or environmental covariance to range from -0.4 to 0.4 (which corresponds to a genetic or environmental correlation ranging from -0.8 to 0.8) in the simulations.

We do understand the reviewer’s concern. Following your comments, we examined heritability as well as genetic and environmental correlations in the real data. Among the 22 examined traits, the heritability estimate ranges from 0.047 to 0.368 with a mean of 0.155; the genetic correlation ranges from -0.731 to 0.945; and the environmental correlation ranges from -0.363 to 0.875. Therefore, the genetic and environmental correlations in our previous simulation settings do closely resemble those observed in the real data. The used heritability of 0.5 in the simulations is higher than that observed in the 22 traits analyzed in the present study, but is clearly not an unreasonable value given that many traits not examined in the present study have a heritability greater than 0.5. Following your comments, we have added additional simulations based on a heritability of 0.15. The details of simulation procedure are provided in Method (lines 263-265 on page 13). The results are largely consistent with our previous simulations with heritability of 0.5. The updated result is shown in S7 Fig with details provided in Results (lines 489-492 on page 23).

**b) The data were simulated from the true model. What if the model is mis-specified? In particular, what if the weights are not optimal? The authors should illustrate that GECKO is robust to misspecification of the model and weights.**

**Response:** Thanks for the comment. Following your comment, we performed new simulations under a mis-specified model where we used weights not obtained in the focused data but from a reference panel. In particular, we used 1,000 genomes project as a reference panel to calculate LD scores so that the weights become mis-specified. The results from the new simulations are largely consistent with the current results, with GECKO performing better than LDSC and GNOVA when the LD pattern does not exactly match the focal population. The simulation details are provided in the Method (lines 266-269 on pages 13), with results shown in Fig S8 and the Results section (lines 492-498 on page 23).

**c) Evaluation of type I error at 0.05 level seems to be too liberal. I am curious to see if GECKO still has controlled type I error rate at a more stringent significance level, e.g. 0.005.**

**Response**: Thanks for the comment. We previously examined type I error at 0.05 using 100 simulation replicates due to the heavy computational burden of all three methods. Following your comment, we have replicated the simulation under the null for 1,000 times and examined type-I error rate at a more stringent significance level of 0.005. Consistent with our previous results, we found that both LDSC and GECKO control for type I error well for covariance testing and produce conservative p-values under a more stringent significance level. GNOVA has an inflated type-I error when there is a presence of sample overlap. The results are shown in the updated Table 1 and mentioned in the Results section (lines 432-447 on pages 20-21).

**2. Real data analysis:**

**a) Please show the heritability for the 22 traits. This will help readers interpret the results and connect with simulations.**

**Response:** Thanks for the comment. The heritability of 22 traits ranges from 0.047 to 0.368 with a mean heritability being 0.155. We included the heritability estimates in the updated Tables S2 and S3, which are cited in the Results (lines 519-520 on page 24).

**b) I am surprised that all estimated covariances are very small. However, even for such small covariances, they are highly significant. For example, the positive genetic covariance between BMI and WBC (covariance = 7.91*10-3; p value =2.24*10-12). What is the biological significance of such a small but highly significant covariance? Can you also show estimation error of the estimated covariances? What I am surprised is that all of the estimated covariances are extremely small. I am not sure that these results are biologically significant. The authors should provide better interpretation of the results and show that these estimates are reliable.**

**Response:** Thanks for the comment. The small genetic covariance obtained from GECKO in the study are actually quite consistent with previous studies (e.g. the LDSC paper). However, we did realize that previous studies have all reported genetic correlation estimates instead of covariance estimates in their real data applications. Therefore, following your comments and the original LDSC and GNOVA papers, we now also report correlation estimates instead of covariance estimates in the updated manuscript. For example, the BMI and WBC example listed in your comment corresponds to a correlation of 0.091. Across 22 traits, the genetic correlation ranges from -0.731 to 0.945 and the environmental correlation ranges from -0.363 to 0.875. All these values are now updated in the Results section.

**c) Since 223 pairs of traits were tested, multiple testing issue should be considered. I am also curious to see the performance of GEKCO at a more stringent significance threshold. Considering correlation among the 223 pairs, 100 independent tests might be a good start. With this, using Bonferroni, the threshold will be 0.05/100=0.0005.**

**Response**: Thanks for the comments. We indeed previously used Bonferroni correction for genetic and environmental covariance testing in the real data, with the p-value threshold for genetic covariance set to be 0.00022 (line 525 on page 24), and the p-value threshold for environmental covariance set to be 0.0009 (line 580 on page 27). We made this point clearer in the updated manuscript (lines 331-332 on page 16).

**3. The methods section should include more details on how the weights are estimated as this is the key of GECKO.**

**Response**: Thanks for the comment. The weight we used is $w_{j}=1/l_{j}$ with $l_{j}$ being the LD score of the *j*’th SNP. The estimation of weights was previously described in the method overview section (currently lines 166-177 on page 9). Following your comments, we have added a few sentences in the compared methods section (lines 309-311 on page 15) to make the weight estimation details clearer.

**4. In terms of comparison, shouldn’t you also compare with mvLMM?**

**Response:** Thanks for the comment. mvLMM requires individual-level genotype data, while GECKO and the currently compared methods (LDSC and GNOVA) only use summary statistics. Consequently, mvLMM cannot be applied to analyze a wide range of data sets that can be analyzed by GECKO, LDSC or GNOVA. Therefore, we did not compare with mvLMM previously. Following your comments, we carried out additional simulations to compare the summary statistics based methods with individual-level data based method mvLMM. As expected, the performance of composite likelihood method GECKO is between the full likelihood based mvLMM and the method of moments based approaches LDSC and GNOVA. The mvLMM fitting details are provided in Method (lines 307-309 on page 15). The results are shown in S4 Fig, with details explained in Results (lines 424-429 on page 20).

**5. Can you comment on the computing time of GECKO? How does it compare with other compared methods with respect to computing speed?**

**Response:** Thanks for the comment. Following your suggestion, we have included computation time for all methods in Results (lines 510-514 on page 24). As expected, GECKO is reasonably efficient though not as fast as the MoM based method like LDSC and GNOVA.

**6. Can you comment on how to use GECKO for admixed populations?**

**Response**: Thanks for the comment. GECKO is not yet directly be applied to admixed population but could be potentially extended by adapting, for example, the framework of cov-LDSC by Luo et al. We added a short discussion on such potential extension in the Discussion (lines 630-639 on page 30).

For a bit more details, we could potentially extend our model in equation (1) (S1 Text) to

$$y_{1}=X_{1}\beta_{1}+\gamma_{1}\eta_{1}+\epsilon_{1},$$

$$y_{2}=X_{2}\beta_{2}+\gamma_{2}\eta_{2}+\epsilon_{2},$$

Where $\eta_{1}$ and $\eta_{2}$ are matrix of top genotype pcs for the study one, and two correspondingly. Followed the idea of cov-LDSC, we could project the covariates out by multiplying $P=I-\eta\left( \eta^{T}\eta\right)^{-1}\eta^{T}=I-\eta\eta^{T}$, we could then get

$$\tilde{y}_{1}=\tilde{x}_{1}\beta_{1}+\tilde{\epsilon}_{1} ,$$

$$\tilde{y}_{2}=\tilde{x}_{2}\beta_{2}+\tilde{\epsilon}_{2},$$

The normality assumption of $\tilde{\epsilon}_{1}$ and $\tilde{\epsilon}_{2}$ are still hold when the sample size is relatively large compared to number of pcs included. The variance of $\tilde{y}_{1}$ and $\tilde{y}_{2}$ should be approximately being one when number of pcs is small compared to number of samples in the study. We could then follow the same derivation procedure for one study setting as shown in equations (1-16) S1 Text (lines 1-106 on pages 1-8). The only difference here is that we use LD score computed $\tilde{x}$, which could be processed using cov-LDSC. The derivations can be tricky in the partially overlapping study and two study design, however, as the same derivations no longer hold unless the two admixed population have the exact same LD structure.

Due to the preliminary nature of the above sketched derivations, we have decided not to include these details in the updated manuscript.

**Minor comments:**

**1. Line 404: How did you compare the power of different methods at fixed type I error rate?**

**Response:** Thanks for the comment. To ensure control at a fixed type I error rate, for each method in turn, we ranked the p-values under the null, obtain the p-value threshold that corresponds to the desired type I error rate, and then used this p-value for the given method as the threshold for power comparison. Therefore, the power of all three methods are controlled at a type-I error rate of 5% instead of a nominal p-value threshold of 0.05. We have included these details in the Method (lines 279-285 on page 14) and briefly mentioned in the Results (line 440-441 on page 21).

**2. Can you comment on why the relative powers of different methods are different across study designs? What is the intuition behind this difference?**

**Response**: Thanks for the comment. The relative power of LDSC and GECKO on detecting non-zero genetic covariance are consistent across different study designs. However, the relative power of GNOVA on detecting non-zero genetic covariance changes with respect to LDSC and GECKO across different designs: it has less powerful than LDSC and GECKO in the one study design and two overlapped study design; while it is more powerful than LDSC and GECKO in the two separate study design. We suspect the main reason underlying the relative power change of GNOVA is due to its implicit assumption on the environmental covariance being zero. Specifically, the fifth equation on page 955 in the GNOVA paper reads

$$\hat{\rho}=M^{-1}v =\frac{\bar{z_{1}z_{2}}}{\bar{r}^{2}\sqrt{n_{1}n_{2}}}.$$

The above equation only holds for the special case of two independent GWAS studies and thus implicitly implies that the environmental covariance is zero. Because the environmental covariance is indeed zero in the two separate study design, GNOVA has higher power than LDSC and GECKO, both of which have to estimate the environmental covariance. We have added a short explanation in the Results (lines 475-481 on pages 22-23).

**3. For real data analysis, it would still be interesting to include GNOVA’s estimates for covariance.**

**Response:** Thanks for the comment. We previously did not include GNOVA in comparison as it does not provide a well-controlled type I error for covariance testing. Following reviewer’s suggestion, we have updated Table S2 to include GNOVA’s estimates.

References

1.	Lu Q, Li B, Ou D, Erlendsdottir M, Powles RL, Jiang T, et al. A Powerful Approach to Estimating Annotation-Stratified Genetic Covariance via GWAS Summary Statistics. Am J Hum Genet. 2017;

2.	Bulik-Sullivan B, Finucane HK, Anttila V, Gusev A, Day FR, Loh PR, et al. An atlas of genetic correlations across human diseases and traits. Nat Genet. 2015;
